# Enhanced In Vitro Antiviral Activity of Hydroxychloroquine Ionic Liquids against SARS-CoV-2

**DOI:** 10.3390/pharmaceutics14040877

**Published:** 2022-04-17

**Authors:** Francisco Faísca, Vanessa Correia, Željko Petrovski, Luís C. Branco, Helena Rebelo-de-Andrade, Miguel M. Santos

**Affiliations:** 1 LAQV-REQUIMTE, Departamento de Química, Faculdade de Ciências e Tecnologia, Universidade Nova de Lisboa, 2829-516 Caparica, Portugal; f.faisca@campus.fct.unl.pt (F.F.); z.petrovski@fct.unl.pt (Ž.P.); l.branco@fct.unl.pt (L.C.B.); 2 Antiviral Resistance Lab, Research & Development Unit, Infectious Diseases Department, Instituto Nacional de Saúde Doutor Ricardo Jorge, IP, Av. Padre Cruz, 1649-016 Lisboa, Portugal; vanessa.correia@insa.min-saude.pt; 3 Host-Pathogen Interaction Unit, Research Institute for Medicines (iMed.ULisboa), Faculty of Pharmacy, Universidade de Lisboa, Av. Professor Gama Pinto, 1649-003 Lisboa, Portugal

**Keywords:** API-ILs, COVID-19, hydroxychloroquine, pharmaceutical ionic liquids, SARS-CoV-2

## Abstract

The development of effective antiviral drugs against SARS-CoV-2 is urgently needed and a global health priority. In light of the initial data regarding the repurposing of hydroxychloroquine (HCQ) to tackle this coronavirus, herein we present a quantitative synthesis and spectroscopic and thermal characterization of seven HCQ room temperature ionic liquids (HCQ-ILs) obtained by direct protonation of the base with two equivalents of organic sulfonic, sulfuric and carboxylic acids of different polarities. Two non-toxic and hydrophilic HCQ-ILs, in particular, [HCQH_2_][C_1_SO_3_]_2_ and [HCQH_2_][GlcCOO]_2_, decreased the virus-induced cytopathic effect by two-fold in comparison with the original drug, [HCQH_2_][SO_4_]. Despite there being no significant differences in viral RNA production between the three compounds, progeny virus production was significantly affected (*p* < 0.05) by [HCQH_2_][GlcCOO]_2_. Overall, the data suggest that the in vitro antiviral activities of the HCQ-ILs are most likely the result of specific intra- and intermolecular interactions and not so much related with their hydrophilic or lipophilic character. This work paves the way for the development of future novel ionic formulations of hydroxychloroquine with enhanced physicochemical properties.

## 1. Introduction

The ongoing coronavirus disease 2019 (COVID-19) pandemic caused by the severe acute respiratory syndrome coronavirus 2 (SARS-CoV-2) has severely affected people’s lives and the well-being of all societies around the world, posing unprecedented challenges to global public health. By mid-April 2022, there were almost 500 million people infected worldwide, of whom more than 6.1 million have died [1]. In addition, the pandemic has triggered a series of economic and social disruptions that have led towards an increase in extreme poverty and inequalities at a global scale, seriously jeopardizing people’s livelihoods for years to come [2].

Despite the approval of limited drug treatments and the emergency use authorization of other drug molecules and monoclonal antibody preparations, antiviral therapy has had still little impact on COVID-19 clinical outcomes for most patients globally [3]. Remdesivir (Veklury^®^), the only antiviral drug approved by both the United States Food and Drug Administration (FDA) and the European Medicines Agency (EMA) for COVID-19 treatment [4,5], has been shown to shorten recovery times in hospitalized patients [6] but to have no effect on either the requirement for ventilation or patient survival [7]. Its intravenous administration also limits its use drastically, particularly with respect to non-hospitalized patients [8]. Developing more effective antiviral molecules against SARS-CoV-2 is hence urgently needed and a global health priority, even more so given the estimated millions of immunocompromised persons who are unlikely to respond robustly to vaccination and the ever-present risk of selection for immune-escape variants that might undermine vaccine effectiveness [3].

With a focus on avoiding the time-consuming stages of drug development, initial attempts to identify efficient drug candidates for COVID-19 were essentially based on drug repurposing efforts wherein existing clinically advanced or marketed drugs were screened for antiviral activity against SARS-CoV-2 in vitro in cell-based infection systems [9]. Chloroquine (CQ) and hydroxychloroquine (HCQ) were two of the promising hits revealed by such screens, showing moderate in vitro activity on Vero E6 cells and inhibiting SARS-CoV-2 infection at both entry and post-entry stages [10,11,12]. Their anti-SARS-CoV-2 activity was in line with the in vitro activity described against past coronaviruses (CoVs) causing human disease outbreaks—SARS-CoV-1 and Middle East respiratory syndrome (MERS) viruses [3].

CQ and HCQ are antimalarial drugs that have also been used in the treatment of autoimmune conditions such as rheumatoid arthritis [13] and systemic lupus erythematosus [14,15,16], in addition to bacterial and viral infections, such as *Coxiella burnetii* infections [17], influenza A and B [18,19], HIV [20] and different versions of human coronavirus [21,22,23]. The mechanism of action by which they accomplish these various effects is, however, not entirely understood [24,25]. The antimalarial activity is attributed to the disruption of lysosomal activity [26,27], while the activity against viral pathogens has been associated with an interference with the glycosylation of ACE2 cellular receptors [28,29,30], lowering the binding affinity to viral spike proteins and preventing cell entry [22,31]. Given their tendency to accumulate within cytoplasmic acidic organelles, including lysosomes and endosomes [32], they might also interfere with endosomal acidification, inhibiting the fusion of viruses to cell membranes and preventing virus entry via the endocytic pathway [33] and, ultimately, certain posterior viral replication processes [34]. Although both drugs share similar chemical structures and mechanisms of action, preference has been given to HCQ due to its lower toxicity.

Notwithstanding the promising in vitro results, treatment of SARS-CoV-2 infection with HCQ did not attain the expected success. Multiple large randomized controlled trials, including the two largest trials—RECOVERY and WHO SOLIDARITY—and open-label trials showed no evidence of clinical benefit from HCQ treatment against SARS-CoV-2 infection; two additional randomized trials showed that HCQ was also ineffective for post-exposure prophylaxis [3]. This lack of clinical efficacy might be explained by HCQ not achieving the required levels of free drug to inhibit SARS-CoV-2 in vivo, even at high dosages, due to its irreversible accumulation in endosomes, Golgi apparatus and lysosomes such that a large portion of the drug is imprisoned, making it inaccessible for action [35].

Therefore, if HCQ could be effective at lower concentrations, with lower cellular accumulation and higher bioavailability, it could find a way to be among the potential antiviral alternatives to fight COVID-19 in a more efficient fashion.

In recent years, the pharmaceutical industry has focused on fewer compounds, due to both increased regulation and a heightened interest in target-specific interactions for the synthesis of newer molecules. This has led to the development of drugs that, despite being active towards their targets, are often insoluble in biological mediums and therefore lack clinical efficiency. This, in turn, has led to a demand for alternative delivery methods. In academia and pharma, some solutions have been investigated regarding such drugs which include the development of prodrugs [36,37], crystal engineering [38,39,40], solid dispersions [41,42], micellar systems [43], organic salts [44] and ionic liquids [45], among others.

Ionic liquids are salts comprised of organic cations and organic or inorganic anions, which melt below 100 °C; if the melting occurs at higher temperatures, the compounds are denominated organic salts. Such organic salts and ionic liquids (OSILs) have been reported to possess surface active properties [46] and also as solubilizing agents of active pharmaceutical ingredients (APIs) [47]. The use of APIs as constituent ions of ILs rendered the third generation of ILs [48], designated API-OSILs or API-ILs according to their melting point. The ionic interactions inherent to this class of compounds have modulated the stability, solubility, bioavailability, pharmacokinetics and biological activity of several APIs [45,49].

In our group, we have developed API-OSILs based on several anionic or cationic drugs, including antibiotics [50,51,52,53,54,55], bone antiresorptive agents [56,57,58] and NSAIDs [59,60]. These can be considered novel ionic formulations of such drugs, which display enhanced water solubility and permeability, as well as reduced polymorphism, thus potentially leading to different pharmacokinetic and delivery profiles. In certain API–counterion combinations, improved biological activity has also been attained [52,53,54,58,61]. In addition, this approach has led to the potential repurposing of some drugs, in particular ampicillin [62] and bisphosphonates [56,57,58], which displayed selective cytotoxic activity against tumor cell lines over normal cells when in the form of API-OSILs.

The dicationic family of ILs [63] typically displays lower toxicity and also higher tuning ability than monocationic ILs, as mixtures of anions with different and/or complementary properties can be used. Hence, very interesting physicochemical [64,65] and biological [66] properties can be achieved.

Therefore, we aimed at preparing a set of novel hydroxychloroquine dicationic ionic liquids as novel formulations of this drug in order to achieve enhanced physicochemical properties and to study their in vitro activity against SARS-CoV-2 in cell lines.

## 2. Materials and Methods

### 2.1. General Remarks

Hydroxychloroquine sulfate (>98.0%) was purchased from TCI Chemicals. Organic acids and salts were purchased from Sigma-Aldrich, TCI Chemicals, Alfa Aesar and Solchemar, and were used as received. The ion-exchange resins Amberlyst A26 (OH) (0.8 meq.mL^−1^ ion-exchange capacity) and Amberlite 15 H^+^ (1.2 meq.mL^−1^ ion-exchange capacity) were purchased from Alfa Aesar and Sigma-Aldrich, respectively. Methanol (PA grade) was acquired from Laborspirit and used as received. The ^1^H and ^13^C NMR spectra of the final compounds were recorded on Bruker Avance II 400 MHz and Avance III 500 MHz spectrometers in DMSO-*d_6_* (Euriso-Top) at 298 K and analyzed using MestreNova. Chemical shifts were reported downfield in parts per million (ppm) from DMSO-*d^6^* references. IR spectra were recorded on a FT-IR Spectrum Two PerkinElmer UATR, 4000–400 cm^−1^ in ATR mode. UV–Visible spectroscopy was performed on a Spectronic Helios Gamma UV–Vis device. DSC analysis was carried out using a Q-series TM Q2000 DSC with a refrigerated cooling system (TA Instruments). The sample was continuously purged with 50 mL/min nitrogen flow. Between 5 and 10 mg of each HCQ-IL were weighed in a standard aluminum pan with a pinhole lid. Elemental analyses were performed by Laboratório de Análises at REQUIMTE, Departamento de Química Faculdade de Ciências e Tecnologia (Monte de Caparica), using a Thermo Finnigan-CE Elemental Analyser 1112 series. High-resolution mass spectrometry (ESI-TOF) was performed at Unidade de Espectrometría de Masas e Proteómica de la Universidade de Santiago de Compostela using a Bruker MicroTOF in FIA mode.

### 2.2. General Procedure for the Synthesis of HCQ-ILs

Hydroxychloroquine sulfate (150 mg, 0.447 mmol) dissolved in a mixture of 1:3 water–methanol was slowly passed through a column packed with Amberlyst A26 (OH) (2.5 mL, 6 equivalents). To the obtained solution of hydroxychloroquine base, two equivalents (0.894 mmol) of the corresponding organic acids (commercially available or prepared by reaction of the corresponding sodium salts with 1.3 mL of Amberlite 15 H^+^) dissolved in distilled water or methanol were added dropwise under stirring at room temperature. After reacting for one hour, the solvent was removed in a rotary evaporator and the pure product was isolated in quantitative yield as a pale-yellow gel after drying under high vacuum for 24 h.

#### 2.2.1. *N*^4^-(7-chloroquinolin-4-yl)-*N*^1^-ethyl-*N*^1^-(2-hydroxyethyl)pentane-1,4-diaminium bis(methanesulfonate), [HCQH_2_][C_1_SO_3_]_2_


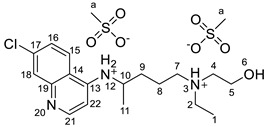


^1^H NMR (400 MHz, DMSO-*d_6_*) δ 8.71 (d, J = 7.7 Hz, 1H, NH-12), 8.67 (d, J = 9.1 Hz, 1H, H-15), 8.54 (d, J = 6.9 Hz, 1H, H-22), 7.94 (d, J = 1.5 Hz, 1H, H-18), 7.75 (dd, J = 9.1 Hz, J = 1.5 Hz, 1H, H-16), 6.94 (d, J = 6.9 Hz, 1H, H-21), 5.31 (br s, 1H, OH-6), 4.14–4.02 (m, 1H, H-10), 3.74–3.67 (m, 2H, H-5), 3.19–3.08 (m, 6H, H-2, H-4, H-7), 2.39 (s, 6H, H-a), 1.87–1.59 (m, 4H, H-8, H-9), 1.31 (d, J = 6.2 Hz, 3H, H-11), 1.18 (t, J = 7.1 Hz, 3H, H-1) ppm. ^13^C NMR (101 MHz, DMSO-*d_6_*) δ 154.8, 143.5, 138.98, 137.9, 126.67, 125.9, 119.4, 115.51, 98.9, 55.2, 53.3, 51.6, 49.2, 48.6, 47.6, 40.2, 31.9, 19.9, 19.5, 8.4 ppm. FTIR-ATR ν 3329, 2970, 1633, 1613, 1576, 1452, 1167, 1039, 771, 551 cm^−1^. HRMS (ESI-TOF) *m*/*z* [M+1]^+^ 336.1839, [M+2]^2+^ 168.5968, [M−1]^−^ 94.9808.

#### 2.2.2. *N*^4^-(7-chloroquinolin-4-yl)-*N*^1^-ethyl-*N*^1^-(2-hydroxyethyl)pentane-1,4-diaminium bis(hexane-1-sulfonate), [HCQH_2_][C_6_SO_3_]_2_


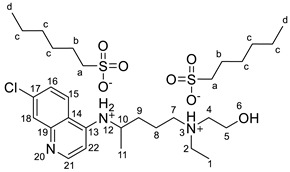


^1^H NMR (400 MHz, DMSO-*d_6_*) δ 8.54 (d, J = 9.1 Hz, 1H, H-15), 8.47 (d, J = 6.3 Hz, 1H, H-22), 8.00 (d, J = 8.0 Hz, 1H, NH-12), 7.87 (d, J = 2.1 Hz, 1H, H-18), 7.62 (dd, J = 9.1, 2.1 Hz, 1H, H-16), 6.76 (d, J = 6.3 Hz, 1H, H-21), 5.28 (br s, 1H, OH-6), 4.01–3.89 (m, 1H, H-10), 3.72–3.65 (m, 2H, H-5), 3.19–3.04 (m, 6H, H-2, H-4, H-7), 2.45–2.36 (m, 4H, H-a), 1.81–1.60 (m, 4H, H-8, H-9), 1.54 (quint, J = 7.5 Hz, 4H, H-b), 1.34–1.20 (m, 15H, H-11, H-c), 1.16 (t, J = 7.2 Hz, 3H, H-11), 0.84 (t, J = 6.8 Hz, 6H, H-d) ppm. ^13^C NMR (101 MHz, DMSO-*d_6_*) δ 152.4, 147.3, 135.8, 125.3, 125.2, 123.1, 116.4, 98.8, 55.3, 53.4, 51.7, 51.5, 48.6, 48.3, 47.5, 32.1, 31.1, 28.1, 25.1, 22.0, 20.1, 19.6, 13.9, 8.5 ppm. FTIR-ATR ν 3402, 3264, 3113, 2957, 2928, 2858, 1634, 1613, 1554, 1151, 1035, 903, 601, 552 cm^−1^. HRMS (ESI-TOF) *m*/*z* [M+1]^+^ 336.1848, [M+2]^2+^ 168.5968, [M−1]^−^ 165.0618.

#### 2.2.3. *N*^4^-(7-chloroquinolin-4-yl)-*N*^1^-ethyl-*N*^1^-(2-hydroxyethyl)pentane-1,4-diaminium bis(1,4-bis((2-ethylhexyl)oxy)-1,4-dioxobutane-2-sulfonate), [HCQH_2_][DocSO_3_]_2_


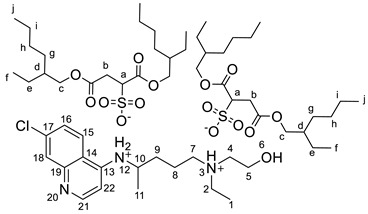


^1^H NMR (500 MHz, DMSO-*d_6_*) δ 8.60 (d, J = 9.1 Hz, 1H, H-15), 8.54 (d, J = 6.8 Hz, 1H, H-22), 8.46 (d, J = 8.2 Hz, 1H, NH-12), 7.90 (d, J = 2.2 Hz, 1H, H-18), 7.74 (dd, J = 9.0, 2.2 Hz, 1H, H-16), 6.89 (d, J = 6.9, 1H, H-21), 5.28 (br s, 1H, OH-6), 4.12–4.00 (m, 1H, H-10), 3.95–3.82 (m, 8H, H-c), 3.72–3.68 (m, 2H, H-5), 3.65 (dd, J = 11.6, 3.6 Hz, 2H, H-a), 3.19–3.06 (m, 6H, H-2, H-4, H-7), 2.92 (dd, J = 17.2, 11.6 Hz, 2H, H-b′), 2.80 (dd, J = 17.2, 3.6 Hz, 2H, H-b″), 1.82–1.60 (m, 4H, H-8, H-9), 1.49 (qt, J = 6.3, 3.2 Hz, 4H, H-d), 1.41–1.20 (m, 35H, H-e, H-g, H-h, H-i, H-11), 1.18 (t, J = 7.2 Hz, 3H, H-1), 0.90–0.79 (m, 24H, H-f, H-j) ppm. ^13^C NMR (126 MHz, DMSO-*d_6_*) δ 171.6, 171.1, 168.3, 153.7, 145.1, 140.8, 137.1, 126.2, 125.5, 120.9, 115.8, 98.8, 66.2, 66.1, 66.1, 66.0, 61.4, 55.1, 53.4, 51.5, 51.5, 48.7, 47.5, 38.2, 38.1, 38.1, 34.1, 33.7, 32.0, 29.7, 29.7, 29.6, 29.5, 28.3, 28.3, 23.1, 23.1, 23.0, 22.9, 22.9, 22.4, 22.3, 19.9, 19.5, 13.9, 13.9, 10.8, 10.7, 10.7, 10.7, 8.4 ppm. FTIR-ATR ν 3266, 2958, 2929, 2860, 1732, 1613, 1459, 1199, 1035, 522 cm^−1^. HRMS (ESI-TOF) *m*/*z* [M+1]^+^ 336.1840, [M+2]^2+^ 168.5966, [M−1]^−^ 421.2276.

#### 2.2.4. *N*^4^-(7-chloroquinolin-4-yl)-*N*^1^-ethyl-*N*^1^-(2-hydroxyethyl)pentane-1,4-diaminium bis(1,4-bis([(1*S*)-7,7-dimethyl-2-oxo-1-bicyclo [2.2.1]heptanyl]methanesulfonic Acid), [HCQH_2_][CampSO_3_]_2_


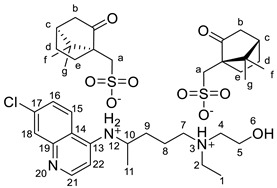


^1^H NMR (500 MHz, DMSO-*d_6_*) δ 8.71 (d, J = 8.2 Hz, 1H, NH-12), 8.66 (d, J = 9.1 Hz, 1H, H-15), 8.53 (d, J = 6.9 Hz, 1H, H-22), 7.95 (d, J = 2.1 Hz, 1H, H-18), 7.75 (dd, J = 9.1, 2.1 Hz, 1H, H-16), 6.93 (d, J = 7.0 Hz, 1H, H-21), 5.29 (br s, 1H, OH-6), 4.14–4.02 (m, 1H, H-10), 3.76–3.64 (m, 2H, H-5), 3.20–3.06 (m, 6H, H-2, H-4, H-7), 2.90 (d, J = 14.8 Hz, 2H, H-a′), 2.73–2.61 (m, 2H, H-c), 2.41 (d, J = 14.8 Hz, 2H, H-a′), 2.23 (dt, J = 18.0, 4.0 Hz, 2H, H-b′, H-b″), 1.93 (t, J = 4.5 Hz, 2H, H-d), 1.90–1.75 (m, 4H, H-b′, H-e′), 1.34–1.21 (m, 7H, H-11, H-d, H-e′, H-e″), 1.17 (t, J = 7.2 Hz, 3H, H-1), 1.04 (s, 6H, H-g), 0.74 (s, 6H, H-f) ppm. ^13^C NMR (126 MHz, DMSO-*d_6_*) δ 216.3, 154.3, 144.1, 139.7, 137.6, 126.4, 125.8, 120.0, 115.6, 98.8, 58.2, 55.2, 53.3, 51.5, 49.0, 48.6, 47.5, 47.0, 46.7, 42.2, 42.1, 31.9, 26.4, 24.1, 20.1, 19.9, 19.5, 8.4 ppm. FTIR-ATR ν 3263, 2955, 1738, 1634, 1613, 1595, 1456, 1152, 1034, 601 cm^−1^. HRMS (ESI-TOF) *m*/*z* [M+1]^+^ 336.1842, [M+2]^2+^ 168.5968, [M−1]^−^ 231.0696.

#### 2.2.5. *N*^4^-(7-chloroquinolin-4-yl)-*N*^1^-ethyl-*N*^1^-(2-hydroxyethyl)pentane-1,4-diaminium bis(4-methylbenzenesulfonate), [HCQH_2_][*p*-TolSO_3_]_2_


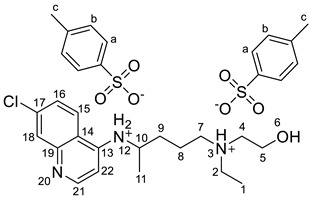


^1^H NMR (400 MHz, DMSO-*d_6_*) δ 8.88 (d, J = 8.1 Hz, 1H, NH-12), 8.67 (d, J = 9.1 Hz, 1H, H-15), 8.56 (d, J = 7.1 Hz, 1H, H-22), 7.94 (d, J = 2.1 Hz, 1H, H-18), 7.74 (dd, J = 9.0, 2.1 Hz, 1H, H-16), 7.51 (d, J = 7.8 Hz, 4H, H-a), 7.13 (d, J = 7.8 Hz, 4H, H-b), 6.97 (d, J = 7.2 Hz, 1H, H-21), 5.31 (br s, 1H, OH-6), 4.17–4.04 (m, 1H, H-10), 3.74–3.66 (m, 2H, H-5), 3.20–3.09 (m, 6H, H-2, H-4, H-7), 2.29 (s, 6H, H-c), 1.72 (d, J = 26.0 Hz, 4H, H-8, H-9), 1.30 (d, J = 6.2 Hz, 3H, H-11), 1.17 (t, J = 7.2 Hz, 3H, H-1) ppm. ^13^C NMR (126 MHz, DMSO-*d_6_*) δ 154.8, 145.3, 143.4, 138.8, 138.0, 137.9, 130.7, 128.8, 128.2, 126.7, 125.9, 125.5, 124.8, 119.3, 115.4, 98.9, 55.2, 53.3, 51.5, 49.2, 48.7, 47.5, 31.9, 20.8, 19.9, 19.6, 8.4 ppm. FTIR-ATR ν 3262, 3112, 3034, 2924, 1633, 1613, 1594, 1457, 1209, 1119, 1164, 1031, 1008, 814, 679, 562 cm^−1^. HRMS (ESI-TOF) *m*/*z* [M+1]^+^ 336.1844, [M+2]^2+^ 168.5976, [M−1]^−^ 171.0137.

#### 2.2.6. *N*^4^-(7-chloroquinolin-4-yl)-*N*^1^-ethyl-*N*^1^-(2-hydroxyethyl)pentane-1,4-diaminium bis(dodecane-1-sulfonate), [HCQH_2_][C_12_SO_4_]_2_


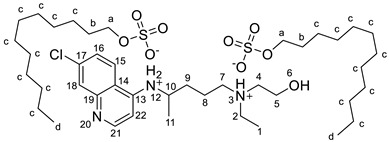


^1^H NMR (400 MHz, DMSO-*d_6_*) δ 8.57 (d, J = 9.1 Hz, 1H, NH-12), 8.53 (d, J = 6.6 Hz, 1H, H-15), 8.30 (d, J = 7.9 Hz, 1H, H-21), 7.89 (d, J = 2.1 Hz, 1H, H-18), 7.72 (dd, J = 9.0, 2.1 Hz, 1H, H-16), 6.86 (d, J = 6.7 Hz, 1H, H-22), 5.26 (br s, 1H, OH-6), 4.01–3.89 (m, 1H, H-10), 3.76–3.61 (m, 6H, H-5, H-a), 3.21–3.04 (m, 6H, H-2, H-4, H-7), 1.89–1.60 (m, 4H, H-8, H-9), 1.47 (q, J = 6.7 Hz, 4H, H-b), 1.30 (d, J = 6.3 Hz, 3H, H-11), 1.29–1.20 (m, 36H, H-c), 1.18 (t, J = 7.2 Hz, 3H, H-1), 0.86 (t, J = 6.6 Hz, 6H, H-d) ppm. ^13^C NMR (126 MHz, DMSO-*d_6_*) δ 206.7, 150.6, 150.2, 147.1, 134.4, 125.8, 124.7, 124.5, 117.1, 98.9, 65.5, 60.7, 55.5, 53.5, 52.9, 51.7, 48.6, 47.7, 47.4, 29.2, 29.1, 29.1, 29.0, 28.8, 28.8, 25.6, 25.6, 22.2, 20.3, 19.8, 14.0, 8.6 ppm. FTIR-ATR ν 3274, 2922, 2853, 1634, 1613, 1578, 1457, 1241, 1195, 1056, 992, 801, 578 cm^−1^. HRMS (ESI-TOF) *m*/*z* [M+1]^+^ 336.1841, [M+2]^2+^ 168.5974, [M−1]^−^ 265.1497.

#### 2.2.7. N^4^-(7-chloroquinolin-4-yl)-N^1^-ethyl-N^1^-(2-hydroxyethyl)pentane-1,4-diaminium bis((2S,3R,4S,5S)-2,3,4,5,6-pentahydroxyhexanoate), [HCQH_2_][GlcCOO]_2_


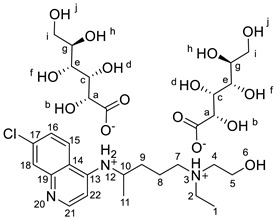


^1^H NMR (400 MHz, DMSO-*d_6_*) δ 8.38 (d, J = 5.2 Hz, 1H, H-15), 8.37 (d, J = 9.1 Hz, 1H, H-21), 7.79 (d, J = 2.3 Hz, 1H, H-18), 7.46 (dd, J = 9.0 Hz, 2.3 Hz, 1H, H-16), 7.02 (d, J = 8.1 Hz, 1H, NH-12), 6.54 (d, J = 5.7 Hz, 1H, H-22), 3.97 (d, J = 3.9 Hz, 2H), 3.90 (dd, J = 3.9 Hz, 2.3, 2H), 3.82–3.70 (m, 1H, H-10), 3.62–3.53 (m, 4H), 3.53–3.42 (m, 4H), 3.36 (m, 2H), 2.86–2.72 (m, 6H, H-2, H-4, H-7), 1.78–1.44 (m, 4H, H-8, H-9), 1.25 (d, J = 6.3 Hz, 3H, H-11), 1.05 (t, J = 7.2 Hz, 3H, H-1) ppm. ^13^C NMR (101 MHz, DMSO-*d_6_*) δ 174.7, 151.5, 149.7, 148.7, 133.5, 127.0, 124.4, 123.9, 117.4, 98.8, 72.7, 72.2, 71.5, 70.5, 63.3, 57.2, 54.4, 52.4, 47.5, 47.4, 32.8, 21.6, 19.7, 9.9 ppm. FTIR-ATR ν 3270, 2933, 1578, 1455, 1403, 1345, 1214, 1080, 1034, 877, 653 cm^−1^. HRMS (ESI-TOF) *m*/*z* [M+1]^+^ 336.1839, [M+2]^2+^ 168.5973, [M−1]^−^ 195.0510.

### 2.3. Water Solubility Studies

The solubility of the hydrophilic HCQ-ILs (with the anions [C_1_SO_3_], [C_6_SO_3_], [CampSO_3_], [*p*-TolSO_3_] and [GlcCOO]) in water was determined by consecutively adding 5 to 10 μL of freshly double-distilled water to an Eppendorf tube containing ca. 30 mg of sample, precisely weighed, until a homogeneous solution was visually observed upon vortex mixing. For the lipophilic HCQ-ILs (with [DocSO_3_] and [C_12_SO_4_] anions), 1 mL volumes of water were added to a falcon tube containing ca. 1 mg of sample, until a maximum of 10 mL was reached.

### 2.4. Octanol–Water Partition Coefficient Studies

The HCQ-ILs’ octanol–water partition coefficients (*K_ow_*) corresponded to the distributions of the synthesized compounds between an aqueous phase and an *n*-octanol organic phase.

A small amount of each HCQ-IL was dissolved in previously prepared octanol-saturated water so as to produce a concentration of 1 mg/mL, and a sample was collected from this solution. To the remaining volume, equal parts of water-saturated octanol were added, followed by 2 h of vigorous stirring. After centrifugation at 5000 rpm for 10 min, a second sample was obtained from the aqueous phase. The two samples were analyzed via UV–Vis spectrophotometry to obtain the initial absorbance (*A_i_*) and the final absorbance (*A_f_*), respectively, in the formula below [67]. Each experiment was performed in triplicate.

(1) $$K_{ow}=\frac{\left(A_{i}\times df_{i}-A_{f}\times df_{f}\right)V_{water}}{A_{f}\times df_{f}\times V_{octanol}}$$

where *K_ow_* = octanol/water partition coefficient, *A_i_* = initial absorbance, *A_f_* = final absorbance, *df* = dilution factor, *V*_water_ = volume of water, *V*_octanol_ = volume of octanol.

For lipophilic HCQ-ILs that failed to dissolve in octanol-saturated water, an analogous procedure was employed using water-saturated octanol to dissolve the compounds. In this context, the *K_ow_* formula was inverted.

### 2.5. Critical Micelle Concentration

The critical micelle concentrations of the HCQ-ILs with surfactant-like anions were calculated using ionic conductivity measurements. A Crison Basic 30+ Radiometer Analytical conductivity meter was used to measure the ionic conductivities (μS/cm) of surfactant solutions in a glass cell at 20 °C containing a magnetic stirrer. For this method, a known amount of Milli-Q water was placed in a thermostated glass cell and the surfactant solution of known concentration was progressively added using a micropipette under constant stirring. Each conductivity value was measured at least three times.

### 2.6. Biological Studies

A preliminary assessment of antiviral activity, based on the capacity to inhibit the virus-induced cytopathic effect (CPE) on treated cells, was initially carried out for the HCQ-ILs, [HCQH_2_][SO_4_] and the corresponding anions as sodium or potassium salts. More comprehensive antiviral activity assays, intended for a direct measurement of SARS-CoV-2 replication (viral RNA transcription and production of infectious progeny viruses), were subsequently performed for the most promising HCQ-ILs. Three independent experiments with triplicate measurements were performed for all assays unless otherwise stated.

All work involving virus propagation and handling of viral cultures was performed in a biosafety level 3 (BLS-3) laboratory, following the WHO recommendations for laboratory biosafety guidance related to the SARS-CoV-2 virus [68].

For cytotoxicity and antiviral activity experiments, [HCQH_2_][SO_4_], most HCQ-ILs and corresponding anions as sodium or potassium salts were dissolved in sterilised water to 1 mM and filtered through a 0.22 μm membrane before being stored at −20 °C in small aliquots as stock solutions. The only exceptions were [HCQH_2_][C_12_SO_4_]_2_ and [HCQH_2_][DocSO_3_]_2_ and the anion of this latter (Na[DocSO_3_]) that were dissolved in dimethyl sulfoxide (DMSO) to 50 mM and directly stored at −20 °C.

### 2.7. Cell Culture

African green monkey kidney Vero E6 cells were obtained from the American Type Culture Collection (ATCC^®^ CRL-1586^TM^) and maintained in Dulbecco’s Modified Eagle Medium (DMEM) supplemented with 2 mM L-glutamine, 1× Non-Essential Amino Acids (NEAA), 24 mM *N*-2-hydroxyethylpiperazine-*N*-2-ethanesulfonic acid (HEPES), 50 μg/mL gentamycin, 2.5 μg/mL fungizone and 10% (*v*/*v*) Fetal Bovine Serum (FBS) at 37 °C in a humidified atmosphere of 5% CO_2_. The culture medium was replaced every 2–3 days and cells were sub-cultured once a week or whenever needed for experiments. All culture reagents were from Gibco, Life Technologies Limited (Paisley, UK).

### 2.8. Virus Stock

SARS-CoV-2 reference strain USA-WA1/2020 (catalog no. NR-52281) was obtained from the Centers for Disease Control and Prevention through the Biodefense and Emerging Infections Research Resources (BEI Resources), the National Institute of Allergy and Infectious Diseases (NIAID) and the National Institutes of Health (NIH) (Manassas, VA, USA) (www.beiresources.org, last accessed on 13 April 2022).

The strain was propagated in culture flasks of confluent Vero E6 cells (4 × 10^4^ cells/cm^2^, cultured overnight) growing in DMEM supplemented with 2 mM L-glutamine, 1× NEAA, 24 mM HEPES, 50 μg/mL gentamycin, 2.5 μg/mL fungizone and 2% (*v*/*v*) FBS (herein designated as DMEM maintenance medium) at 37 °C in a 5% CO_2_ atmosphere. Cultures were observed daily, and the virus was harvested when 80–90% of the cells manifested CPE. After the second passage, cell supernatants were collected and centrifuged at 4 °C 3000 rpm for 10 min to remove cell debris and stored at −80 °C in small aliquots as a working stock.

Stock viral titer was then determined by 50% Tissue Culture Infectious Dose (TCID_50_) assay, using 10 replicates for each serial 10-fold dilution (from 10^−1^ to 10^−8^). Briefly, confluent Vero E6 monolayers (1.25 × 10^4^ cells/well) cultured overnight in clear flat-bottom 96-well plates were infected with serial 10-fold dilutions of virus stock formerly prepared in DMEM supplemented with 2 mM L-glutamine, 1× NEAA and 24 mM HEPES (herein designated as DMEM base medium). After 1h of incubation at 37 °C (5% CO_2_), the cells were washed with Dulbecco’s phosphate-buffered saline (DPBS) (Gibco, Life Technologies Limited, Paisley, UK) and cultured with fresh DMEM maintenance medium for 72 h at previous incubation conditions. Virus-induced CPE was recorded under an inverted optical microscope, and viral titer, expressed as TCID_50_/mL, was calculated following the method of Reed and Müench [69].

### 2.9. Cell Viability Assay

The CellTiter-Glo^®^ Luminescent Cell Viability Assay (Promega, Madison, WI, USA) was used to measure the cytotoxic effects of the different compounds (HCQ-ILs and parental drug) and anions (as sodium or potassium salts) on Vero E6 cells. Briefly, confluent Vero E6 monolayers (1.25 × 10^4^ cells/well) cultured overnight in white-wall clear-bottom 96-well plates were incubated with serial 2- and 10-fold dilutions (0.5 μM to 400 μM) of the compounds in DMEM maintenance medium at 37 °C and 5% CO_2_. Non-toxic and vehicle assay control wells were instead treated with equal volumes of, respectively, oseltamivir carboxylate (OSC; F. Hoffmann-La Roche Ltd., Basel, Switzerland; 2-fold serial dilutions—0.16 μM to 20 μM) and DMEM maintenance medium alone or with DMSO (0.8%). Wells with only vehicle medium (no cells) were used as background control. After 72 h, CellTiter-Glo^®^ Reagent was added to plates according to manufacturer’s instructions, and luminescence was recorded using a FLUOstar OPTIMA plate reader (BMG Labtech, Madrid, Spain). A 10× lysis solution (Promega) was added to half of the vehicle-treated cells (2 times triplicate) 30 min before adding the assay reagent to include a positive toxic control in the assay.

Raw luminescence values, expressed in relative light units (RLUs), were analysed in a spreadsheet to calculate the percentage of cell viability relative to vehicle control and determine the 50% cytotoxic concentration (CC_50_) (i.e., the concentration that reduces cell viability by 50%) of each compound and counterion by point-to-point curve fitting.

### 2.10. Antiviral Activity Assays

#### 2.10.1. Inhibition of Virus-Induced CPE

Vero E6 cells (1.25 × 10^4^ cells/well) cultured overnight in white-wall clear-bottom 96-well plates were pre-treated with serial non-toxic concentrations of the compounds and anions as sodium/potassium salts (0.1 μM to 25 μM) prepared in DMEM base medium. After 1 h incubation at 37 °C (5% CO_2_), cells were challenged with SARS-CoV-2 virus at 100 TCID_50_ and returned to the incubator for another 1 h at the same conditions. Fresh DMEM maintenance medium containing the indicated concentrations of compounds/anions was added after washing cell monolayers twice with DPBS, and plates were incubated for 72 h at standard conditions (37 °C, 5% CO_2_). DMEM maintenance medium alone or with DMSO (0.05%) was instead added to virus, vehicle and background (no cells) assay control wells. Virus-induced CPE was measured by CellTiter-Glo^®^ Luminescent Cell Viability Assay, following the manufacturer’s instructions and using a Tecan Infinite M200 plate reader (TECAN, Männedorf, Switzerland) for recording luminescence.

Raw luminescence values were analysed in a spreadsheet to calculate the percentage of CPE inhibition relative to virus control (% = 100 × ((RLU infected treated cells − mean RLU virus control)/(mean RLU vehicle control − mean RLU virus control)); and determine the 50% (EC_50_) and 90% (EC_90_) effective concentration (i.e., the concentration that inhibits virus-induced CPE by 50% and 90%, respectively) of each compound by point-to-point curve fitting. Selectivity indexes (SI) were calculated from the relationship between CC_50_ and EC_50_. Only two independent experiments were performed for the anions.

#### 2.10.2. Inhibition of Viral RNA Transcription and Infectious Progeny Production

The pre-treatment, infection and treatment of cell monolayers were performed as described above for CPE inhibition, with only minor differences that included: the 96-well plates used (clear flat-bottom); the concentration range tested (1 μM to 50 μM); and the extent of final incubation (48 h). After 48 h post-infection (hpi), the cell supernatants of the three replicates under identical conditions were collected into the same tube and centrifuged at 3000 rpm for 10 min to remove the cell debris before being stored at −80 °C in small aliquots. Viral RNA (vRNA) transcription was assessed by quantification of virus yield in cell supernatants using a quantitative real-time RT-PCR (qRT-PCR) assay, while a TCID_50_ assay was used to evaluate the production of infectious progeny viruses by determination of the infectious virus titer in the equivalent supernatants. Only qRT-PCR is detailed below, since the TCID_50_ assay was performed as described above for virus stock titration. Data from both assays were analysed in a spreadsheet to calculate the percentage of inhibition relative to virus control and to determine the EC_50_, EC_90_ and SI relative to each parameter as described for CPE inhibition. Four independent experiments were performed, as an additional assay was needed to clarify qRT-PCR results.

##### 2.10.3. qRT-PCR Assay

Viral RNA was extracted from 140 μL of cell supernatant using a QIAamp^®^ Viral RNA Mini Kit (QIAGEN, Hilden, Germany) according to the manufacturer’s instructions and stored at −80 °C until it was used. Quantitative analysis of purified RNA was performed using the in-house real-time RT-PCR protocol described by Skittrall [70], targeting a 222-base region within the RNA-dependent RNA polymerase (RdRp) region of the SARS-CoV-2 1ab gene. A standard curve was generated using duplicate serial dilutions (10^8^–10^1^) of the SARS-CoV-2 NSP12 VersaClone cDNA plasmid (R&D Systems, Minneapolis, MN, USA; catalog no. RDC3140), propagated in TOP10 Chemically Competent *E. coli* cells (Invitrogen, Thermo Fisher Scientific, Carlsbad, CA, USA) and quantified in an Eppendorf BioPhotometer (Hamburg, Germany) (ηg/μL) after purification using a QIAprep Spin Miniprep Kit (QIAGEN) according to the manufacturer’s instructions. Reactions were run on an Applied Biosystems 7500 Real-Time PCR machine (Applied Biosystems, Foster City, CA, USA).

### 2.11. Statistical Analysis

Graphical representations and statistical analyses were performed using GraphPad Prism software version 9.2.0 for Mac (GraphPad Software, San Diego, CA, USA). Significant differences in CC_50_, EC_50_ and EC_90_ values were evaluated using a one-way analysis of variance (ANOVA) followed by Tukey’s multiple comparison test. A *p*-value < 0.05 was considered significant.

## 3. Results and Discussion

We describe the synthesis of seven new ionic liquids containing hydroxychloroquine (HCQ-ILs) as dications by direct protonation with two equivalents of biocompatible anions. All prepared HCQ-ILs were characterized by spectroscopic techniques and their solubility in water and octanol–water partition coefficients were determined. The critical micelle concentrations of the most lipophilic HCQ-ILs were also determined. Lastly, evaluation of cytotoxicity and anti-SARS-CoV-2 activity in Vero E6 cells was performed.

### 3.1. Synthesis and Characterization

**Scheme 1:**
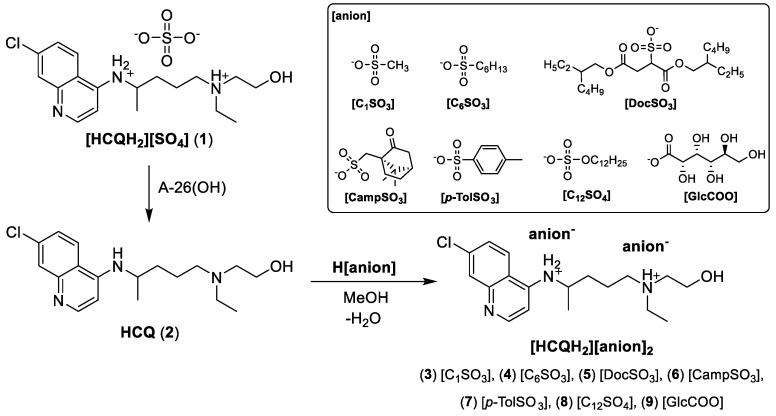
Methodology for the synthesis of the HCQ-ILs.

The synthesis of the HCQ-ILs was performed by the dropwise addition of the selected organic acids to a hydroxychloroquine base (HCQ), in an analogous fashion to previous works with other drugs (e.g., fluoroquinolones [51]). HCQ was previously prepared by passing hydroxychloroquine sulfate ([HCQH_2_][SO_4_], **1**), the commercial form of HCQ, through the hydroxide exchange resin Amberlyst A-26 (OH). The obtained HCQ free base (**2**) was subsequently protonated at the alkyl secondary and tertiary amines in aqueous media with two equivalents of five sulfonic acids, one sulfuric acid and one carboxylic acid based on the following anions: methanesulfonate [C_1_SO_3_] (**3**), hexanesulfonate [C_6_SO_3_] (**4**), docusate [DocSO_3_] (**5**), (1*S*)-(-)-10-camphorsulfonate [CampSO_3_] (**6**), *p*-toluenesulfonate [*p*-TolSO_3_] (**7**), dodecylsulfate [C_12_SO_4_] (**8**) and gluconate [GlcCOO] (**9**). Some of the organic acids were prepared from the corresponding sodium salts by previously undergoing ion exchange via the cationic resin Amberlite 15(H^+^). Scheme 1 shows the employed synthetic methodology.

All HCQ-ILs were isolated as gels and hence considered as room temperature ionic liquids (RTILs). They were characterized by FTIR and NMR (^1^H, ^13^C) spectroscopies, high-resolution mass spectrometry (ESI-TOF) as well as elemental analysis. The thermal properties were studied by differential scanning calorimetry, and the critical micelle concentration of the most lipophilic compounds (**5** and **8**) were studied by electric conductivity measurements.

**Figure 1:**
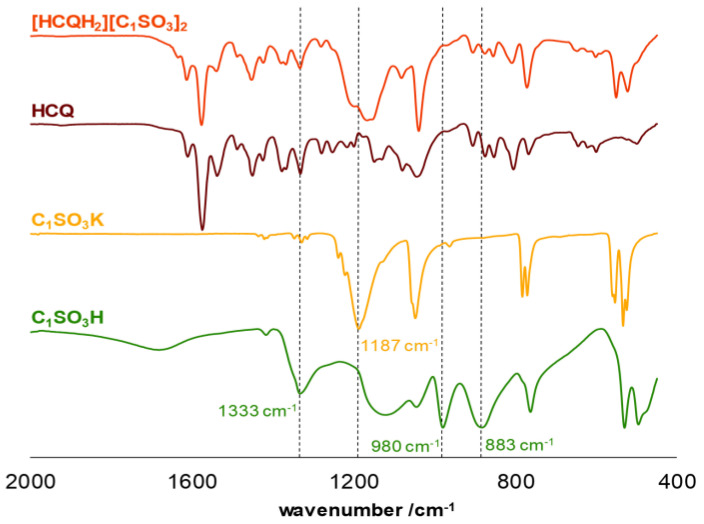
FTIR-ATR spectra of [HCQH_2_][C_1_SO_3_]_2_ (**3**), HCQ (**2**), C_1_SO_3_K and C_1_SO_3_H between ν 2000–400 cm^−1^.

The FTIR spectra of the prepared HCQ-ILs (Figures S1–S7) supported the full ionization of the organic acids. In the case of compound **9**, the presence of a carboxylate stretching band at 1578 cm^−1^ and the absence of one at ca. 1730 cm^−1^, corresponding to the stretching of the carboxylic acid group, confirms the complete ionization of this HCQ-IL. In the case of the sulfonate-containing ILs **3**–**7**, similar vibrational profiles within the 1350–850 cm^−1^ fingerprint zone were observed between the starting anion salts and corresponding products, which were strikingly different for HCQ and corresponding acids. Hence, this corroborated the presence of the anions and cations as ionized species. Figure 1 depicts a comparison of the FTIR spectra of [HCQH_2_][C_1_SO_3_]_2_ (**3**), hydroxychloroquine (**2**), potassium methanesulfonate (C_1_SO_3_K) and also methanesulfonic acid (C_1_SO_3_H). In more detail, the bands at 1333, 980 and 883 cm^−1^ are characteristic of non-ionized methanesulfonic acid, as they are absent from the spectra of the remaining compounds. In addition to the absence of these bands, the FTIR spectra of product **3** contains a band that appears at 1187 cm^−1^ in the spectrum of potassium methanesulfonate, ascribable to the stretching of the sulfonate group. Hence, these data indicate the ionization of methanesulfonic acid upon combination with hydroxychloroquine.

Regarding the ^1^H NMR spectra (Figures S8–S21), all presented only one set of signals in the intended HCQ–anion stoichiometry of 1.0:2.0. Comparison with the ^1^H NMR spectrum of the original drug (**1**) confirmed the structural integrity of HCQ upon the reaction. Furthermore, all ^13^C NMR spectra contain the expected signals from both the anions and the cation.

In all cases, high-resolution mass spectra in positive and negative modes (Figures S22–S35) confirmed the presence of hydroxychloroquine cations and the corresponding anions, respectively. On the one hand, the positive mode presented both the [M+1]^+^ and [M+2]^2+^ peaks, correspondingly at an average *m*/*z* 336.1842 (−0.89 ≤ error ≤ −3.27 ppm) and *m*/*z* 168.5970 (−7.71 ≤ error ≤ −12.46 ppm) (Table S1). On the other hand, the negative mode spectra displayed the peak corresponding to [M−1]^-^ at the expected *m*/*z* values, with an absolute error comprehended between −2.61 and −16.96 ppm (Table S2). The observed isotopic distribution was as expected for each case.

### 3.2. Thermal Properties

As previously mentioned, all HCQ-ILs were obtained as room temperature ionic liquids, as opposed to the starting [HCQH_2_][SO_4_], which is a solid that melts at 245 °C (Figure S36). Each sample was studied by differential scanning calorimetry (Figures S37–S43) by sequentially heating and cooling the sample between −90 and 100 °C at 10 °C/min (two cycles) and 20 °C/min (one final cycle). One preliminary isotherm at 100 °C for 10 min was performed to remove residual water. In agreement with the amorphous state of the compounds, all thermograms displayed one glass transition temperature (T_g_), which was calculated in the last heating cycle (Table 1).

In addition, none of the compounds presented a tendency for crystallization in the several cooling cycles performed.

These data suggest that T_g_ has a decreasing trend with increasing length of the sulfonate/sulfate alkyl chain. More specifically, the HCQ-IL with the smallest sulfonate anion, [C_1_SO_3_] (**3**), presented a T_g_ of 29.9 °C, which decreased to 9.9 for [C_6_SO_3_] (**4**) and subsequently to −7.9 and −11.4 in the combinations with [DocSO_3_] (**5**) and [C_12_SO_4_] (**8**), respectively. This may be attributable to a more disorganized arrangement of the vitreous state in the HCQ-ILs bearing long alkyl chain anions.

### 3.3. Water Solubility and Octanol–Water Partition Coefficient Studies

On the one hand, the solubility of the prepared HCQ-ILs was determined by adding known volumes of freshly double-distilled water to fixed amounts of compounds at 37 °C until complete dissolution was observed by visual inspection. On the other hand, the octanol–water partition coefficients were measured by preparing ca. 1 mg/mL solutions of each HCQ-IL in octanol-saturated water, which were then thoroughly stirred with an equal volume of water-saturated octanol. UV–Visible absorption spectra were recorded for the initial and final aqueous or organic solutions, depending on the solubility of each HCQ-IL in the solvents. The results are presented as the logarithm of *K_ow_*, Log P. Table 2 compiles the data obtained from both experiments.

As expected, most HCQ-ILs are more soluble in water than the original drug (84 mg/mL), with the exception of the ones containing the lipophilic anions [DocSO_3_] (**5**) and [C_12_SO_4_] (**8**). While the latter are insoluble in water (lower than 0.5 mg/mL), the former displayed very high solubility comprehended between 910 ([C_6_SO_3_], **4**) and 2020 mg/mL ([CampSO_3_], **6**). Accordingly, the water-soluble compounds display lower Log P values than the water-insoluble ones. However, the correlation is not linear. In the particular case of [HCQH_2_][C_1_SO_3_]_2_ (**3**), it showed the second highest solubility and also a Log P value near zero, consistent with only a slight preference for aqueous media in the presence of an apolar phase. The remaining salts displayed similar Log P values, independently of their water solubility profiles. While the latter followed the trend **6** > **7** > **9** > **4** with very different recorded values, their Log P followed a different trend of **4** > **7** > **6** > **9**, nonetheless with very similar values.

Hence, the most soluble salts would rapidly and extensively dissolve after oral administration, which could mean that they have a higher oral bioavailability than the least soluble ones. In addition, positive or near positive Log P values may also lead to a higher degree of interaction with apolar structures, such as cellular membranes, which could also account for a potentially high drug uptake. An optimal balance between hydrophilic and lipophilic properties is required in order to enhance hydroxychloroquine oral bioavailability and consequently its therapeutic activity, while modulating its excretion and distribution throughout many different tissues and organs in order to reduce systemic toxicity.

### 3.4. Critical Micelle Concentration

The ability to form micelles of the lipophilic HCQ-ILs [DocSO_3_] (**5**) and [C_12_SO_4_] (**8**) was studied by ionic conductivity measurements. The inherent ability of these HCQ-ILs to self-aggregate into micelles can render adequate drug delivery properties. By measuring the conductivity of solutions with increasing concentrations of the compounds, two distinct slopes were observed for each case (see Figures S44 and S45 in ESI), consistent with the formation of micelles. The critical micelle concentration (cmc) values, calculated by resolving both equations, are given in Table 3, alongside the cmc values for the starting halide salts (Na[DocSO_3_] and [C_12_SO_4_]).

As expected, the cmc values of the starting halide salts are much higher (*ca.* three orders of magnitude) than those for the HCQ-ILs due to the higher degree of hydration of the former, which hinders the process of micelle formation, and hence higher concentrations of compounds are required. On the other hand, by adsorbing into the micellar surface in an easier fashion, the less hydrated HCQ-ILs decrease the charge repulsion between the polar heads and micelles are formed at lower concentrations.

### 3.5. Cytotoxicity in Vero E6 Cells

**Figure 2:**
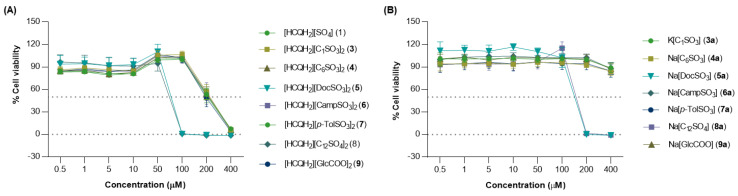
Comparative cytotoxicity of the (**A**) different compounds (HCQ-ILs and parental drug) and (**B**) counterions in non-infected Vero E6 cells, evaluated by the amount of ATP present in cultures (CellTiter-Glo^®^ Luminescent Cell Viability Assay) and expressed in relative values compared to vehicle control (DMEM maintenance medium alone or with 0.8% DMSO). Data are plotted as mean percent values across three independent experiments with triplicate measurements. The error bars represent the standard deviation of the mean (note: some error bars are shorter than the height of the symbol). Graphical representations were generated using GraphPad Prism software version 9.2.0 for Mac (GraphPad Software, San Diego, CA, USA).

In order to assess the biocompatibility of the compounds under study, cytotoxicity assays were performed on Vero E6 cells, which are the gold standard for SARS-CoV-2 propagation studies. As shown in Figure 2A, the majority of the HCQ-ILs displayed a similar cytotoxic profile to the parental drug, possessing CC_50_ values ranging from 196.0 to 218.7 μM (CC_50_ (**1**) = 214.4 μM) (Table 4).

The only exceptions to this biocompatible profile were the lipophilic HCQ-ILs **5** and **8**, which exhibited CC_50_ values of 69.8 and 73.2 μM, respectively. This behaviour was supported by the high toxicity exhibited by the corresponding lipophilic anions **5a** and **8a**, the only ones found to be toxic to these cells, as evidenced in Figure 2B, with CC_50_ values of 142.2 μM and 148.9 μM, respectively (Table 4).

In other words, all HCQ-ILs were considered biocompatible with the exception of the latter two, which were found to be ca. three times more toxic than the original molecule in vitro. The enhanced lipophilic properties of these HCQ-ILs could promote disruption of the cell membrane and possibly lead to drug accumulation inside the cell beyond safety levels.

### 3.6. In Vitro Antiviral Efficacy against SARS-CoV-2

#### 3.6.1. Inhibitory Effects on Virus-Induced CPE

**Figure 3:**
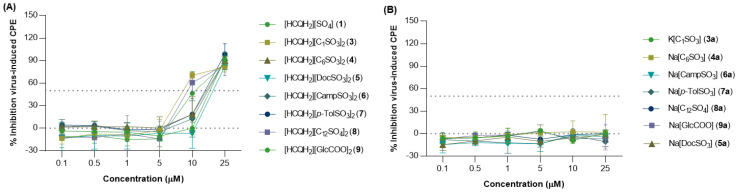
Inhibition of SARS-CoV-2 replication based on virus-induced cytopathic effect (CPE) at 72 h post-infection (hpi) in Vero E6 cells treated with serial non-toxic concentrations of compounds (**A**) or anion salts (**B**). CPE was measured by calculating the number of live cells via a CellTiter-Glo^®^ Luminescent Cell Viability Assay. Results are expressed in relation to virus control (untreated infected cells). Data are plotted as mean percent values across three and two independent experiments with triplicate measurements, respectively. Data were plotted and generated as indicated in Figure 2.

Treatment of cell monolayers with [HCQH_2_][C_1_SO_3_]_2_ (**3**), [HCQH_2_][C_12_SO_4_]_2_ (**8**) and [HCQH_2_][GlcCOO]_2_ (**9**) resulted in more than 60% inhibition of CPE at 10 μM (70.5%, 60.8% and 62.5%, respectively), which contrasted with the remaining compounds, including **1** (lower than 20%; see Figure 3A).

The EC_50_ values of these three novel formulations (8.1, 8.9 and 8.5 μM, respectively) were significantly lower than those of all other HCQ-ILs and differed by ca. two-fold from the EC_50_ of **1** (16.5 μM) (Table 5). No significant differences in EC_90_ were observed between the seven novel HCQ-ILs and **1**, with all values being registered beyond 20 μM.

**Figure 4:**
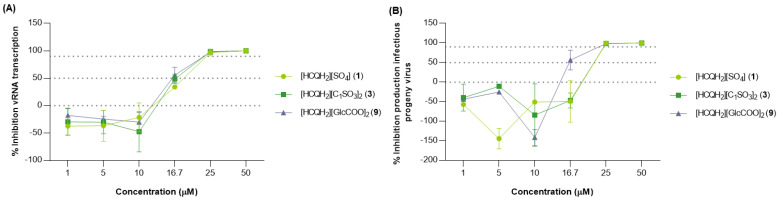
Dose–response curves for the two HCQ-OSILs identified as promising and for the parental drug (**1**) based on viral RNA (vRNA) transcription (**A**) and progeny production (**B**). RNA transcription was assessed by quantification of virus yield using qRT-PCR assay (RdRp gene) and progeny production was determined by calculation of the infectious virus titer by TCID_50_ assay (TCID_50_/mL), both at 48 hpi in cell supernatants. Results are expressed in relative values compared to virus control. Data were plotted and generated as indicated in Figure 2. Four independent experiments were carried out on vRNA transcription assays (**A**).

The enhanced antiviral activity of **3** and **9** in this initial screening doubled their SI ratio (26.9 and 23.1, respectively) in comparison with **1** (13.0) (Table 5), leading them to be identified as the most promising HCQ-ILs. Hence, these were selected for further and more comprehensive evaluation of their anti-SARS-CoV-2 activity by direct measurement of inhibition of virus infection (see Section 3.6.2). Despite its high activity, **8** (Ec_50_ 8.9 μM) presented one of the lowest SI ratios (7.8) given its high cytotoxicity towards Vero E6 cells (CC_50_ 69.8 μM) and was thus not selected for the subsequent inhibition studies. Also of note, none of the anions showed antiviral activity against SARS-CoV-2 (Figure 4B).

#### 3.6.2. Inhibitory Effects on vRNA Transcription and Progeny Production

The two most promising formulations, **3** and **9**, were evaluated, together with the parental drug, for their capacity to inhibit vRNA transcription and the production of infectious progeny. In the case of **9**, over 50% inhibition in both experiments was observed at 16.7 μM (55.8% and 56.5%, respectively; Figure 4A,B).

On the other hand, **3** exhibited inhibitory profiles closer to the original molecule, causing a slightly lower inhibition (48.5%; **1**: 34.0%) of vRNA transcription (Figure 4A) and no further inhibition of progeny production at the same concentration (Figure 4B). In fact, **9** presented very similar EC_50_ values for both experiments (16.3 and 16.9 μM, respectively), and these were found to be the lowest for the three compounds. From both of these values, only the one regarding progeny production differed significantly from the EC_50_ values of **3** (21.8 μM) and the parental drug **1** (21.7 μM). No significant differences in EC_90_ values were observed among compounds for either of the read-outs, with values varying between 22 μM and 25 μM (Table 6). The SI ratios of both HCQ-ILs for the two read-outs were found to be slightly higher than that of the original molecule, as evidenced in Table 6.

These data indicate that there was a slightly significant (*p* < 0.05) enhanced inhibition of the production of virus particles capable of causing infection in the presence of **9** that was not observed for **3** and the parental drug **1**. However, there was no significant difference in the production of viral RNA (from both infectious and non-infectious viruses) among the three compounds. These parameters yield a more robust estimation of the compounds’ impact on virus infection than the one based on the inhibition of the virus-induced CPE, as in the latter case there was only an estimation based on the differentiation between viable and non-viable (dead) cells in the presence of SARS-CoV-2.

## 4. Conclusions

Since none of the anions (as sodium or potassium salts) possessed antiviral activity against the studied strain of SARS-CoV-2, the observed enhanced activities of the two most promising HCQ-ILs, [HCQH_2_][C_1_SO_3_]_2_ (**3**) and [HCQH_2_][GlcCOO]_2_ (**9**), are suggestive of specific intramolecular (between cation and anions) and intermolecular (between the HCQ-ILs, cell organelles and/or viral structural components) interactions. Moreover, these activities seem not to correlate with the lipophilic or hydrophilic properties of the compounds. On one hand, both **3** and **9** possess water-solubility and Log P values similar to other tested HCQ-ILs that did not show improved antiviral activity. On the other hand, the highly lipophilic [HCQH_2_][DocSO_3_] (**5**) showed CPE inhibition similar to the two promising hydrophilic HCQ-ILs, while the analogously apolar [HCQH_2_][C_12_SO_4_]_2_ (**8**) was even less effective in inhibiting the virus-induced CPE than the original drug. This work paves the way for the development of ionic formulations of hydroxychloroquine with enhanced physicochemical properties.

## Figures and Tables

**Table 1:** Glass transition temperatures of the seven HCQ-based RTILs.

HCQ-ILs	T_g_/°C
[HCQH_2_][C_1_SO_3_]_2_ (**3**)	29.9
[HCQH_2_][C_6_SO_3_]_2_ (**4**)	9.9
[HCQH_2_][DocSO_3_]_2_ (**5**)	−7.9
[HCQH_2_][CampSO_3_]_2_ (**6**)	43.0
[HCQH_2_][*p*-TolSO_3_]_2_ (**7**)	26.3
[HCQH_2_][C_12_SO_4_]_2_ (**8**)	−11.4
[HCQH_2_][GlcCOO]_2_ (**9**)	0.4

**Table 2:** Water solubility (in mg/mL) and Log P values of the prepared HCQ-ILs.

HCQ-ILs	Water Solubility (mg/mL)	Log P
[HCQH_2_][SO_4_] (**1**)	84	−2.25
[HCQH_2_][C_1_SO_3_]_2_ (**3**)	1800	−0.22
[HCQH_2_][C_6_SO_3_]_2_ (**4**)	910	−1.04
[HCQH_2_][DocSO_3_]_2_ (**5**)	<0.5	1.24
[HCQH_2_][CampSO_3_]_2_ (**6**)	2020	−1.16
[HCQH_2_][*p*-TolSO_3_]_2_ (**7**)	1400	−1.12
[HCQH_2_][C_12_SO_4_]_2_ (**8**)	<0.5	1.01
[HCQH_2_][GlcCOO]_2_ (**9**)	1230	−1.39

**Table 3:** Critical micelle concentrations (mol/L) of **5**, **8** and corresponding sodium anion salts.

HCQ-ILs	cmc/M	Reference cmc/M (25 °C)
[HCQH_2_][DocSO_3_]_2_ (**5**)	1.79 × 10^−6^	-
Na[DocSO_3_] (**5a**)	1.54 × 10^−3^	5.0 × 10^−3^ [71]
[HCQH_2_][C_12_SO_4_]_2_ (**8**)	4.83 × 10^−6^	-
Na[C_12_SO_4_] (**8a**)	9.60 × 10^−3^	8.0 × 10^−3^ [72]

**Table 4:** Half-maximal cytotoxic activity (CC_50_) of [HCQH_2_][SO_4_] (**1**), HCQ-ILs and corresponding anions on Vero E6 cells. Results are presented as the mean ± standard deviation of three independent experiments with triplicate measurements. Significant (SIG) differences were evaluated using a one-way analysis of variance (ANOVA) followed by Tukey’s multiple comparison test. A *p*-value < 0.05 was considered significant, with *** *p* < 0.001 and **** *p* < 0.0001.

Compounds	CC_50_ (μM)	95% CI	SIG Differences
[HCQH_2_][SO_4_] (**1**)	214.4	148.7–280.1	
[HCQH_2_][C_1_SO_3_]_2_ (**3**)	218.7	157.1–280.2	
[HCQH_2_][C_6_SO_3_]_2_ (**4**)	214.3	154.9–273.7	
[HCQH_2_][DocSO_3_]_2_ (**5**)	69.8	63.0–76.5	**** **1**,**3**,**4**,**7**,**8**,**9**
[HCQH_2_][CampSO_3_]_2_ (**6**)	73.2	68.0–78.5	**** **1**,**3**,**4**,**7**,**8** *** **9**
[HCQH_2_][*p*-TolSO_3_]_2_ (**7**)	206.7	142.4–270.9	
[HCQH_2_][C_12_SO_4_]_2_ (**8**)	209.3	116.0–302.6	
[HCQH_2_][GlcCOO]_2_ (**9**)	196.0	134.8–257.1	
K[C_1_SO_3_]_2_ (**3a**)	>400	-	
Na[C_6_SO_3_]_2_ (**4a**)	>400	-	
Na[DocSO_3_]_2_ (**5a**)	148.9	141.2–156.5	**** **3a**,**4a**,**7a**,**8a**,**9a**
Na[CampSO_3_]_2_ (**6a**)	142.2	127.3–157.1	**** **3a**,**4a**,**7a**,**8a**,**9a**
Na[*p*-TolSO_3_]_2_ (**7a**)	>400	-	
Na[C_12_SO_4_]_2_ (**8a**)	>400	-	
Na[GlcCOO]_2_ (**9a**)	>400	-	

**Table 5:** The 50% (EC_50_) and 90% (EC_90_) effective concentrations of [HCQH_2_][SO_4_] (**1**) and HCQ-ILs **3–9** for the inhibition of the virus-induced cytopathic effect (CPE) on Vero E6 cells infected with SARS-CoV-2. The results are presented as the mean of three independent experiments with triplicate measurements. The 95% confidence interval (CI) is indicated in a separate column. SI represents the CC_50_/EC_50_ selectivity ratio. No inhibitory effects were found for the anions. Significant differences (SIG diff.) were evaluated using a one-way analysis of variance (ANOVA) followed by Tukey’s multiple comparison test. A *p*-value < 0.05 was considered significant, with * *p* < 0.05, ** *p* < 0.01, *** *p* < 0.001.

Compounds	EC_50_ (μM)	95% CI	SIG Diff.	EC_90_ (μM)	95% CI	SIG Diff.	SI
[HCQH_2_][SO_4_] (**1**)	16.5	13.2–19.7		23.0	20.9–25.2		13.0
[HCQH_2_][C_1_SO_3_]_2_ (**3**)	8.14	7.0–9.3	* **4**; ** **1**,**7**,**8**; *** **6**	>25	-		26.9
[HCQH_2_][C_6_SO_3_]_2_ (**4**)	14.4	7.1–21.7		24.2	21.2–27.1		14.9
[HCQH_2_][DocSO_3_]_2_ (**5**)	8.9	4.9–12.9	* **4**,**7**; ** **1**,**8**; *** **6**	20.9	20.3–21.5	* **4**,**8**; ** **3**,**6**,**9**	7.8
[HCQH_2_][CampSO_3_]_2_ (**6**)	17.8	13.3–22.2		>25	-		4.1
[HCQH_2_][*p*-TolSO_3_]_2_ (**7**)	15.0	9.3–20.7		21.1	13.6–28.5	** **3**,**6**,**9**	13.8
[HCQH_2_][C_12_SO_4_]_2_ (**8**)	15.8	11.3–20.3		23.8	21.7–26.0		13.3
[HCQH_2_][GlcCOO]_2_ (**9**)	8.5	6.8–10.2	* **4**,**7**; ** **1**,**6**,**8**;	>25	-		23.1

**Table 6:** The 50% (EC_50_) and 90% (EC_90_) effective concentrations of [HCQH_2_][SO_4_] and HCQ-ILs for the inhibition of viral RNA (vRNA) transcription and production of progeny infectious viruses on Vero E6 cells infected with SARS-CoV-2. The results are presented as the mean of four (vRNA) and three (progeny) independent experiments with triplicate measurements. The 95% confidence interval (CI) is indicated in a separate column. SI represents the CC_50_/EC_50_ selectivity ratio. Significant differences (SIG diff.) were evaluated using a one-way analysis of variance (ANOVA) followed by Tukey´s multiple comparison test. A * *p*-value < 0.05 was considered significant.

Compounds	Inhibition of vRNA Transcription					
	EC_50_ (μM)	95% CI	SIG Diff.	EC_90_ (μM)	95% CI	SI
[HCQH_2_][SO_4_] (**1**)	18.6	17.7–19.4		25.1	18.4–31.8	11.6
[HCQH_2_][C_1_SO_3_]_2_ (**3**)	17.0	15.3–18.8		23.4	23.2–23.6	12.8
[HCQH_2_][GlcCOO]_2_ (**9**)	16.3	13.9–18.7		23.1	21.3–24.9	12.0
	**Inhibition of production of infectious progeny viruses**					
[HCQH_2_][SO_4_] (**1**)	21.7	19.2–24.2		24.3	21.7–26.9	9.9
[HCQH_2_][C_1_SO_3_]_2_ (**3**)	21.8	21.1–22.6		24.4	24.1–24.7	10.0
[HCQH_2_][GlcCOO]_2_ (**9**)	16.9	12.4–21.4	* **1**,**3**	22.5	18.7–26.3	11.6

## Data Availability

Not applicable.

## Supplementary Materials

The following supporting information can be downloaded at: https://www.mdpi.com/article/10.3390/pharmaceutics14040877/s1, Figures S1–S7: FTIR spectra of the HCQ-ILs, Figures S8–S21: ^1^H and ^13^C APT NMR spectra of the HCQ-ILs, Figures S22–S35: ESI-TOF mass spectra in positive and negative modes of the HCQ-ILs, Figures S36–S43: DSC thermograms of the HCQ-ILs, Figures S44 and S45: Plots of conductivity vs. concentration of **5** and **8**, Tables S1–S2: Experimental and calculated *m*/*z* values and corresponding errors (ppm) for the [M+1]^+^, [M+2]^2+^ and [M–1]^−^ ions of the ESI-TOF spectra for each HCQ-OSIL.

## Abbreviations

API-ILs: Active pharmaceutical ingredient ionic liquids; API-OSILs, active pharmaceutical ingredient ionic liquids and organic salts; C_12_SO_4_, dodecylsulfate; C_1_SO_3_, methanesulfonate; C_6_SO_3_, hexanesulfonate; CC_50_, half-maximal cytotoxic concentration; cmc, critical micelle concentration; CPE, cytopathic effect; CampSO_3_, (1*S*)-(-)-10-camphorsulfonate; DocSO_3_, docusate; EC_50_, half-maximal effective concentration; EC_90_, 90% effective concentration; GlcCOO, gluconate; HCQ, hydroxychloroquine; HCQ-ILs, hydroxychloroquine ionic liquids; IL, ionic liquid; *K_ow_*, octanol–water partition coefficient; Log P, logarithm of *K_ow_*; RTILs, room temperature ionic liquids; T_g_, glass transition temperature; *p*-TolSO_3_, *p*-toluenosulfonate; vRNA, viral ribonucleic acid.
