# Supplementary material for: Enhanced In Vitro Antiviral Activity of Hydroxychloroquine Ionic Liquids against SARS-CoV-2
Source: Pharmaceutics. 2022 Apr 17;14(4):877. doi: 10.3390/pharmaceutics14040877 (PMC9031298; doi:10.3390/pharmaceutics14040877)
Supplement: Supplementary file 1 [file pharmaceutics-14-00877-s001.zip › pharmaceutics-1633087-supplementary.pdf]

Supporting Information

Enhanced In Vitro Antiviral Activity of Hydroxychloroquine Ionic Liquids Against SARS-CoV-2

FTIR spectra

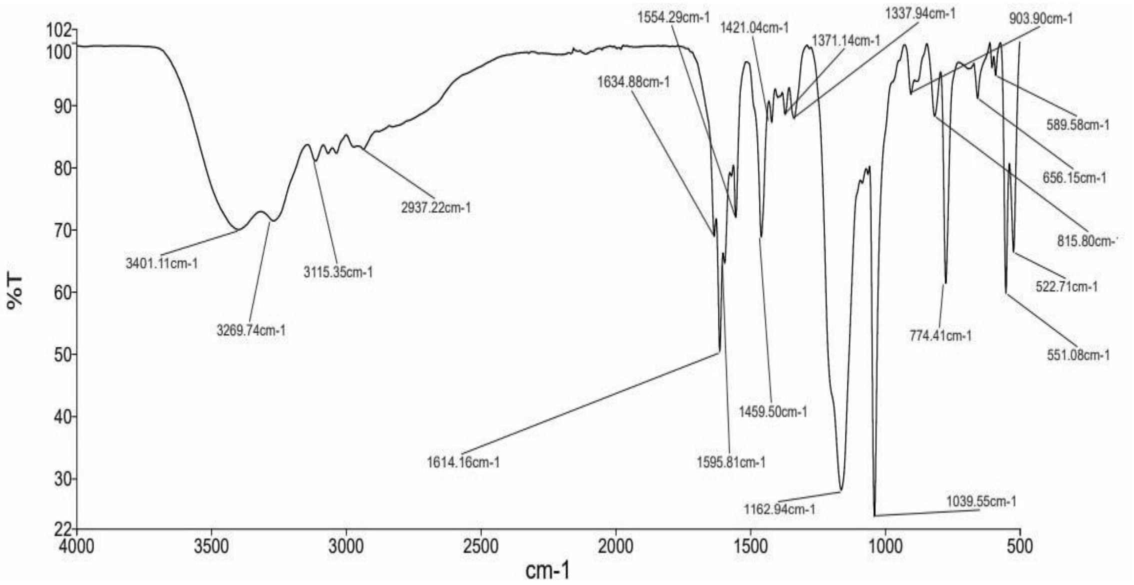

Figure S1. FTIR-ATR spectrum of $[HCQH_2][C_1SO_3]_2$.

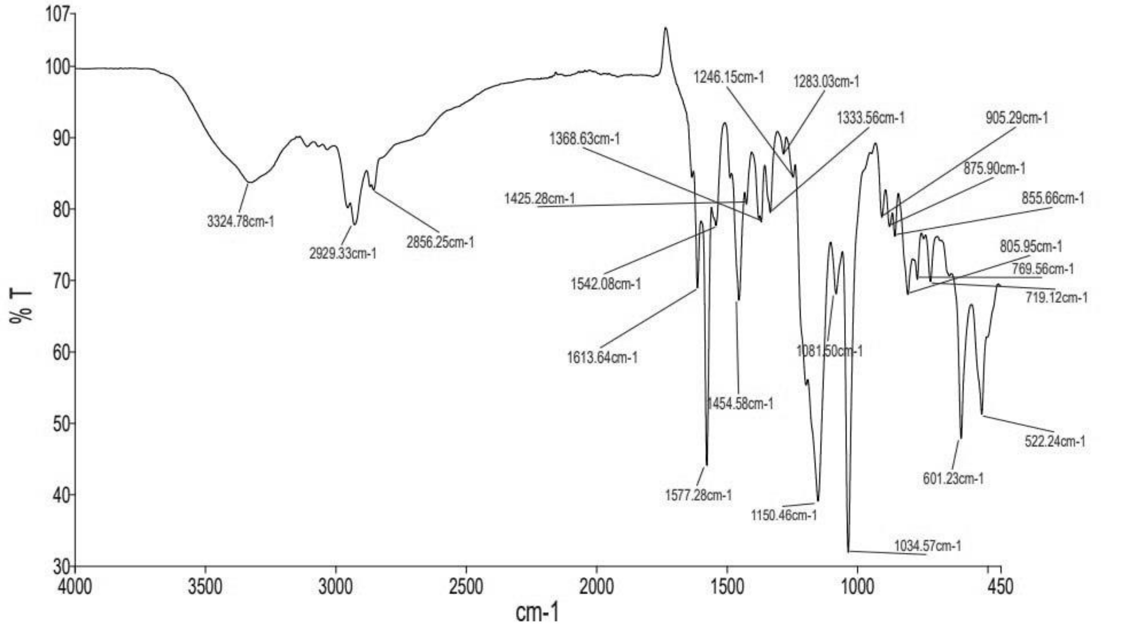

Figure S2. FTIR-ATR spectrum of $[HCQH_2][C_6SO_3]_2$.

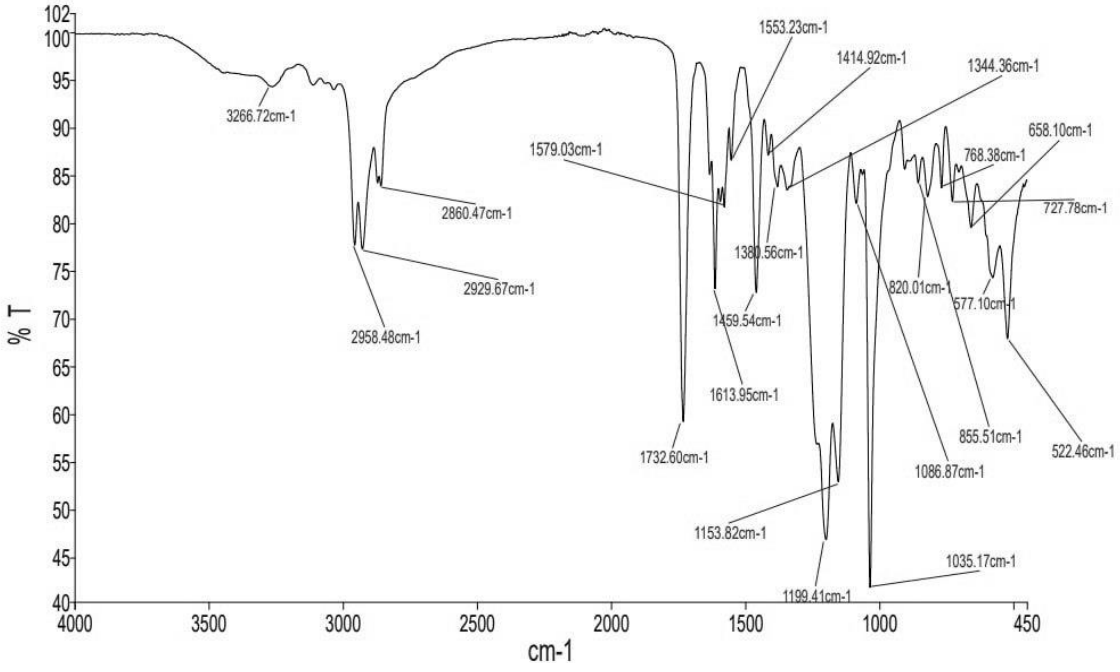

Figure S3. FTIR-ATR spectrum of $[HCQH_2][DocSO_3]_2$.

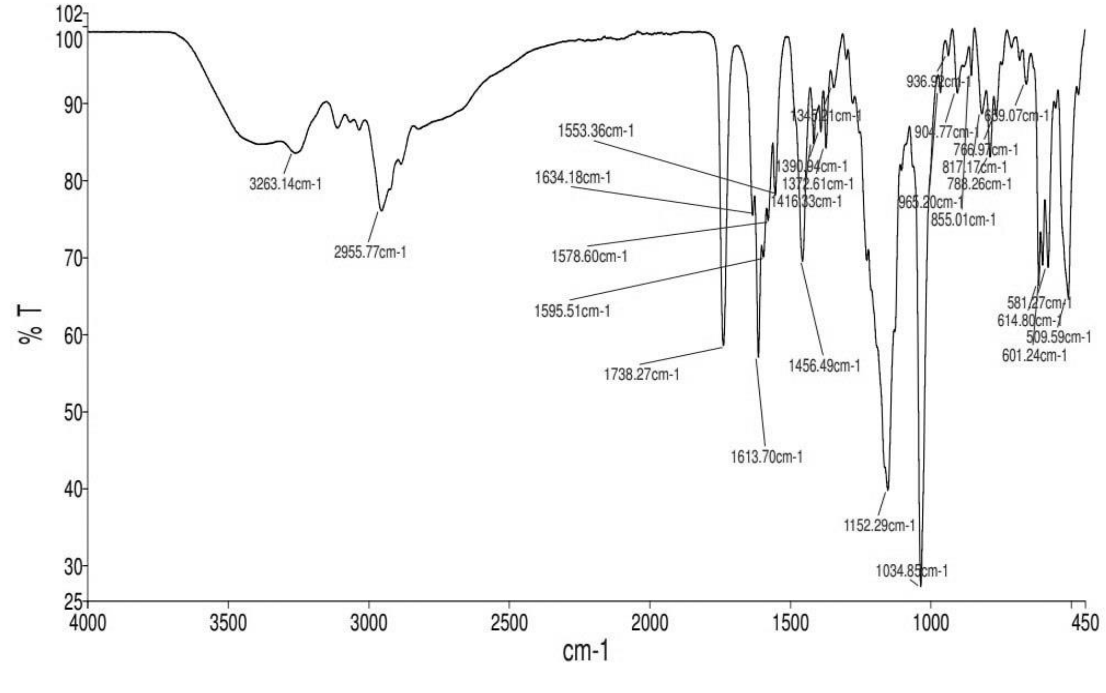

Figure S4. FTIR-ATR spectrum of $[HCQH_2][CampSO_3]_2$.

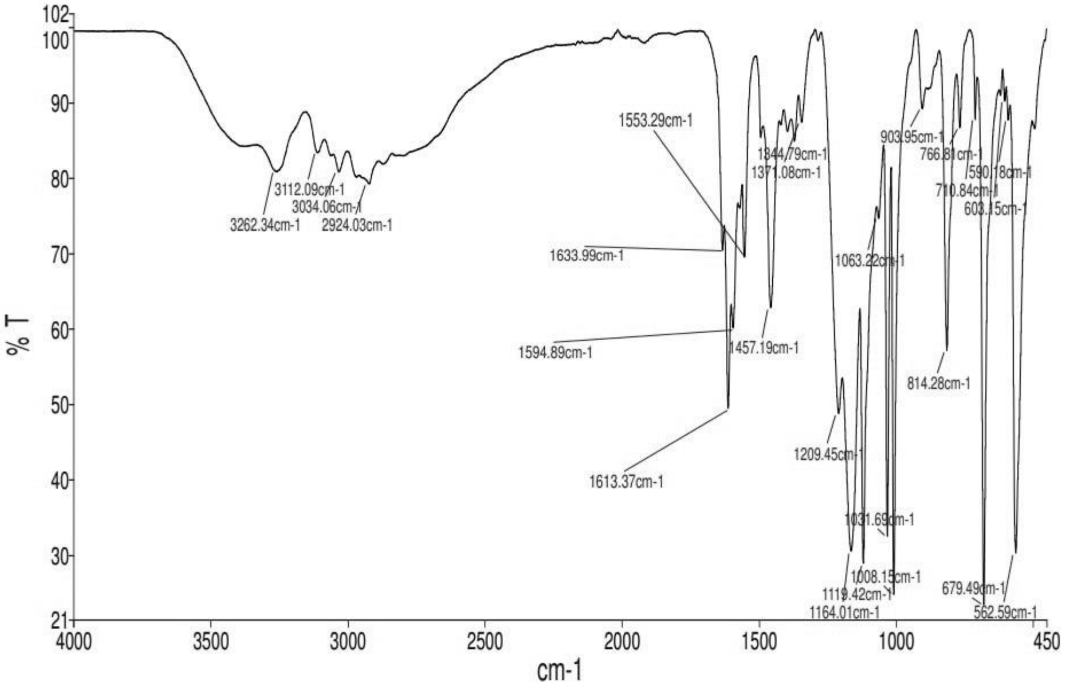

Figure S5. FTIR-ATR spectrum of $[HCQH_2][p\text{-}TolSO_3]_2$.

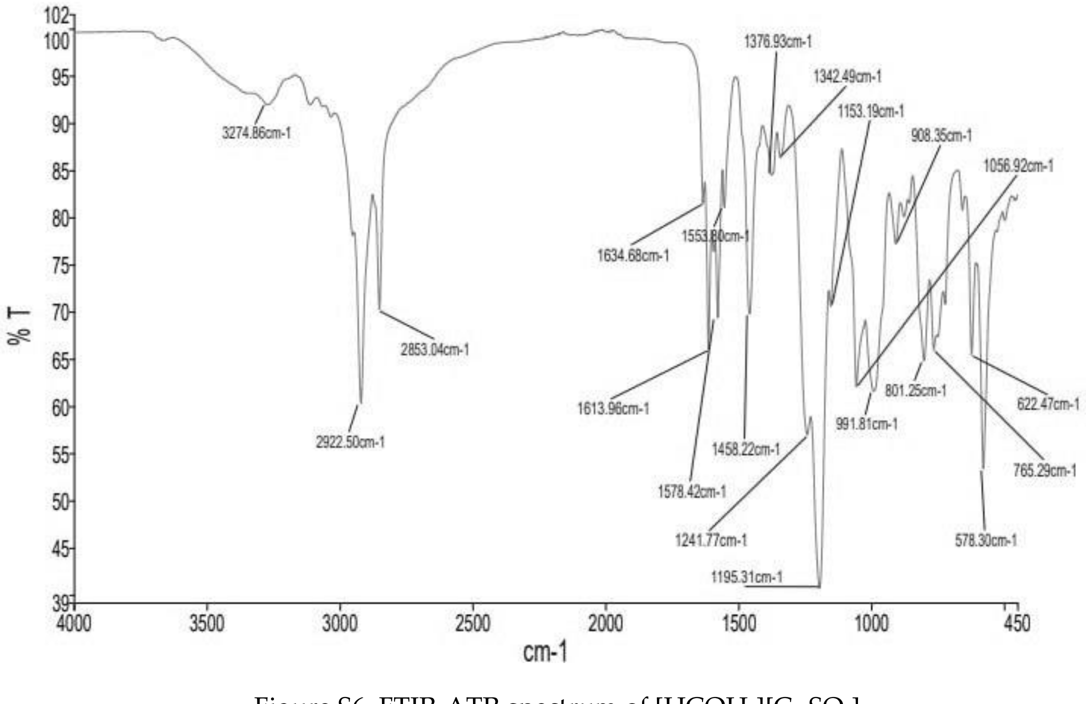

Figure S6. FTIR-ATR spectrum of $[HCQH_2][C_{12}SO_4]_2$.

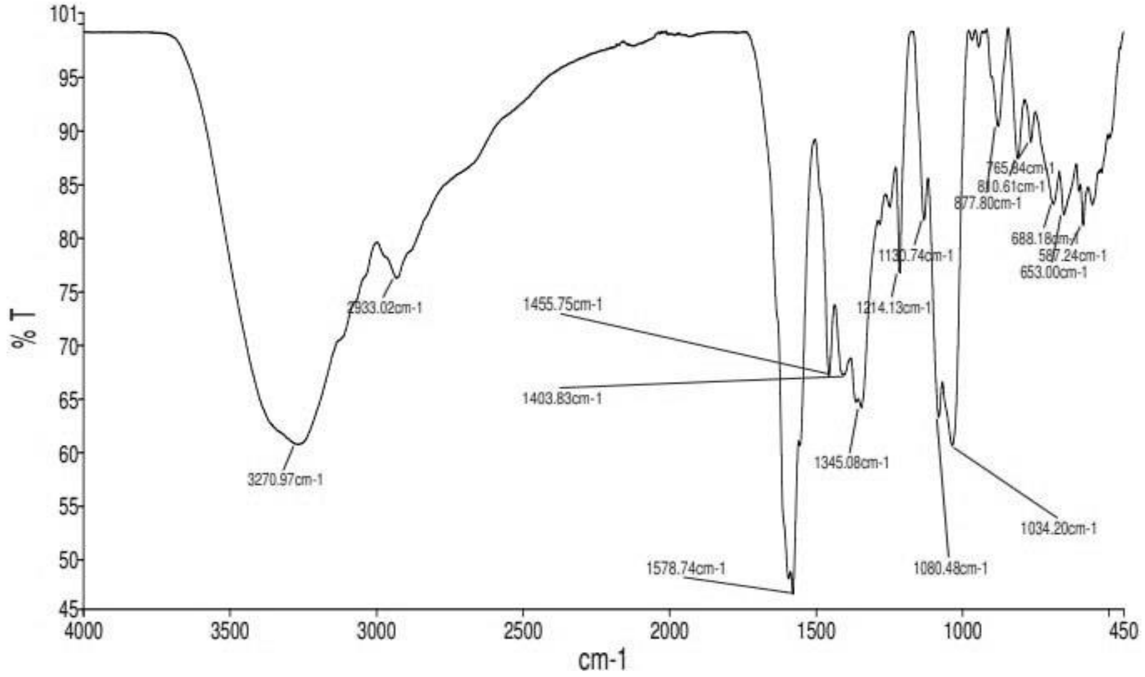

Figure S7. FTIR-ATR spectrum of [HCQH2][GlcCOO]2.

NMR spectra

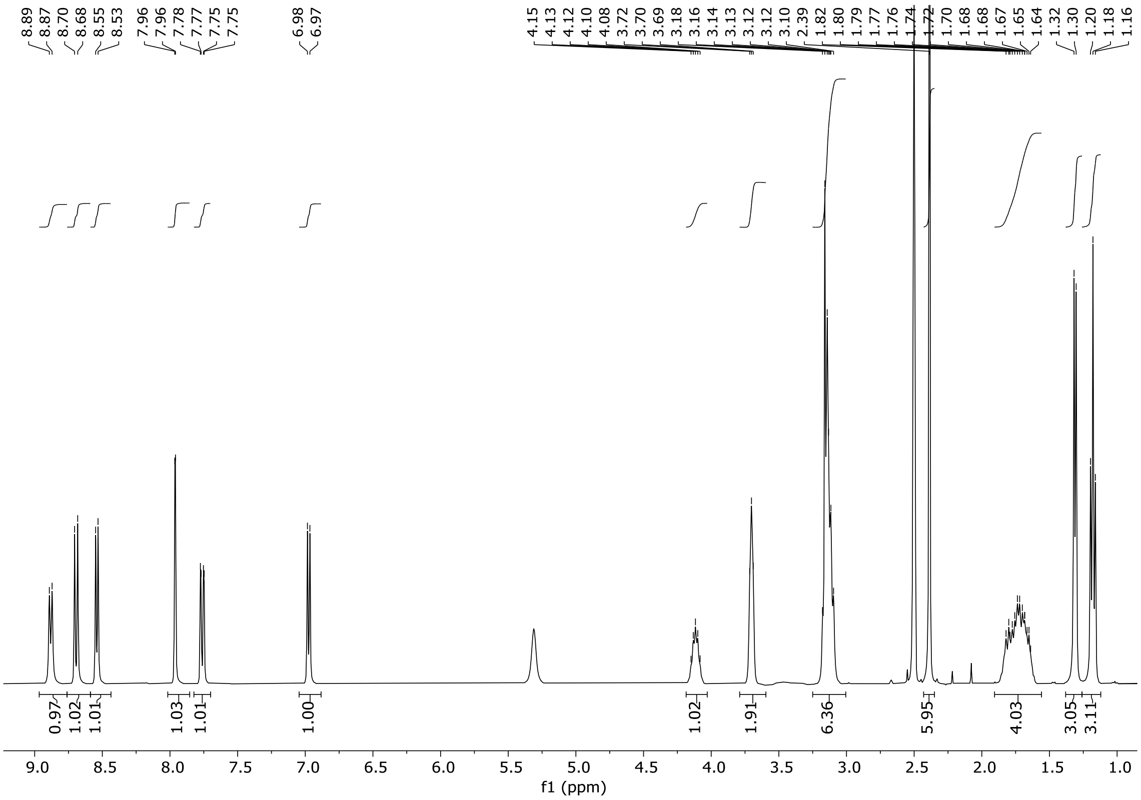

Figure S8. ^{1}H NMR spectrum of [HCQH$_2$][C$_1$SO$_3$]$_2$ in DMSO-d_6.

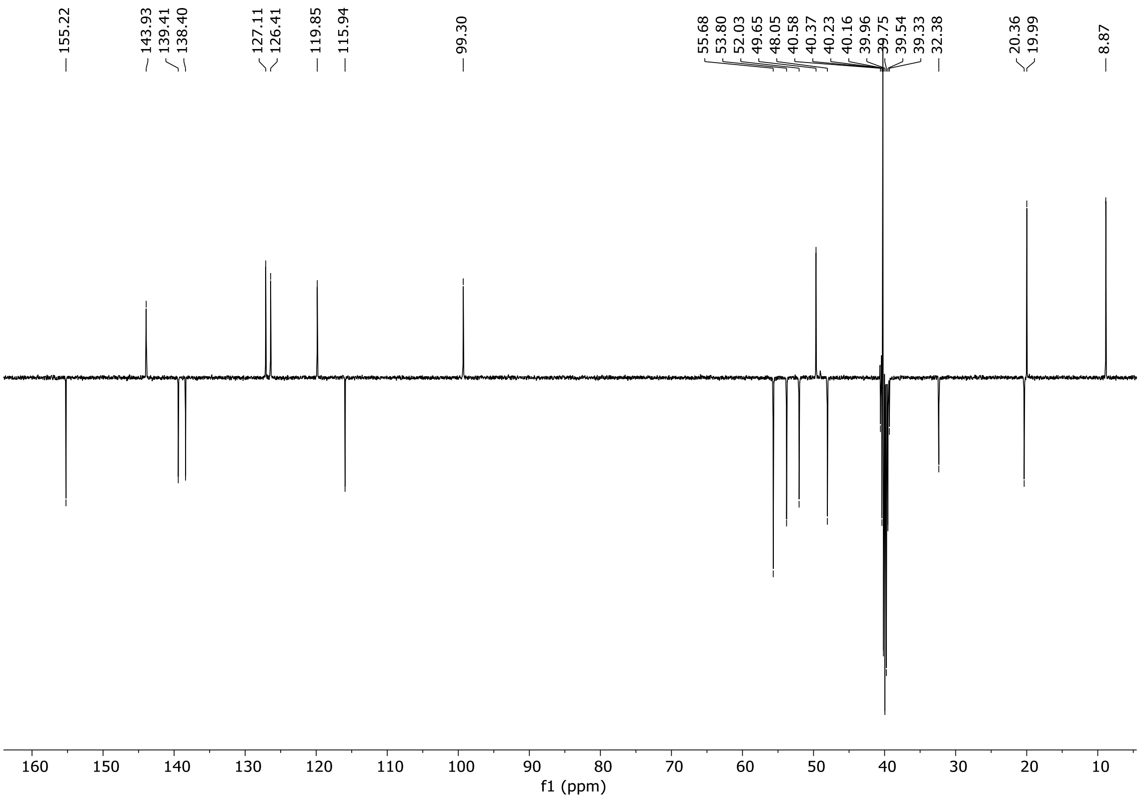

Figure S9. ^{13}C APT NMR spectrum of [HCQH$_2$][C$_1$SO$_3$]$_2$ in DMSO-d_6.

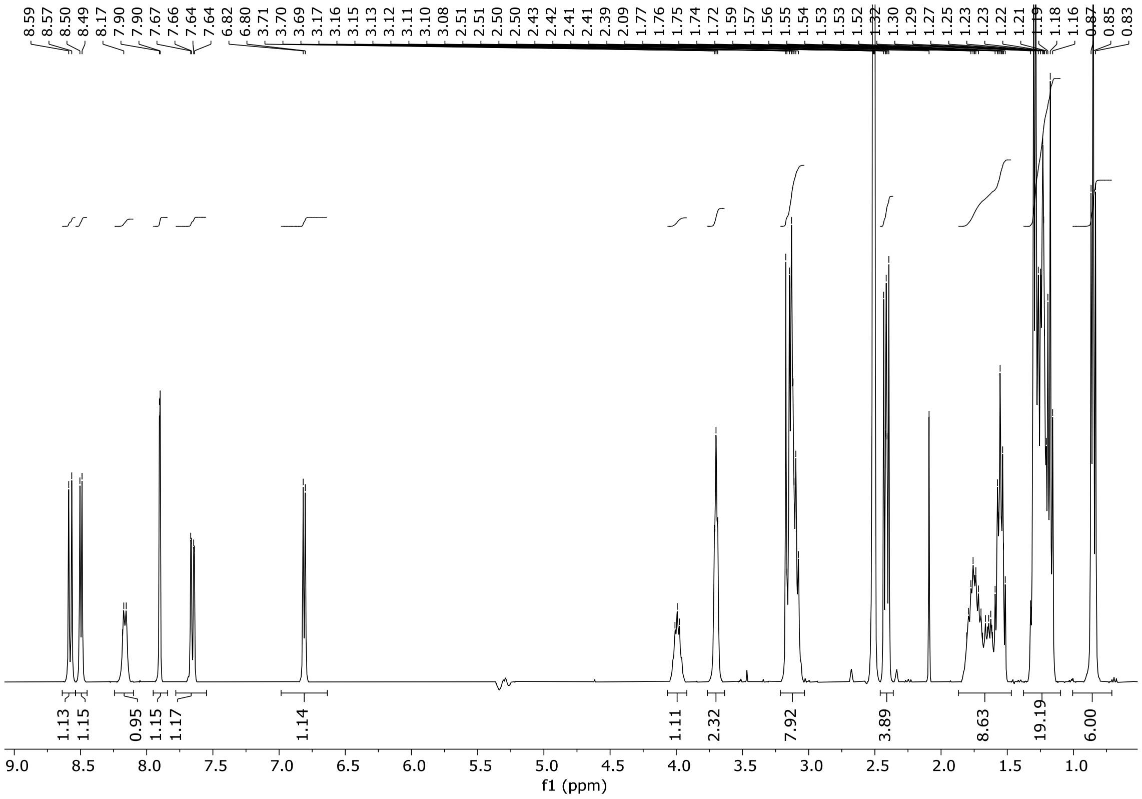

Figure S10. ^{1}H NMR spectrum of $[HCQH_2][C_6SO_3]_2$ in DMSO-d_6.

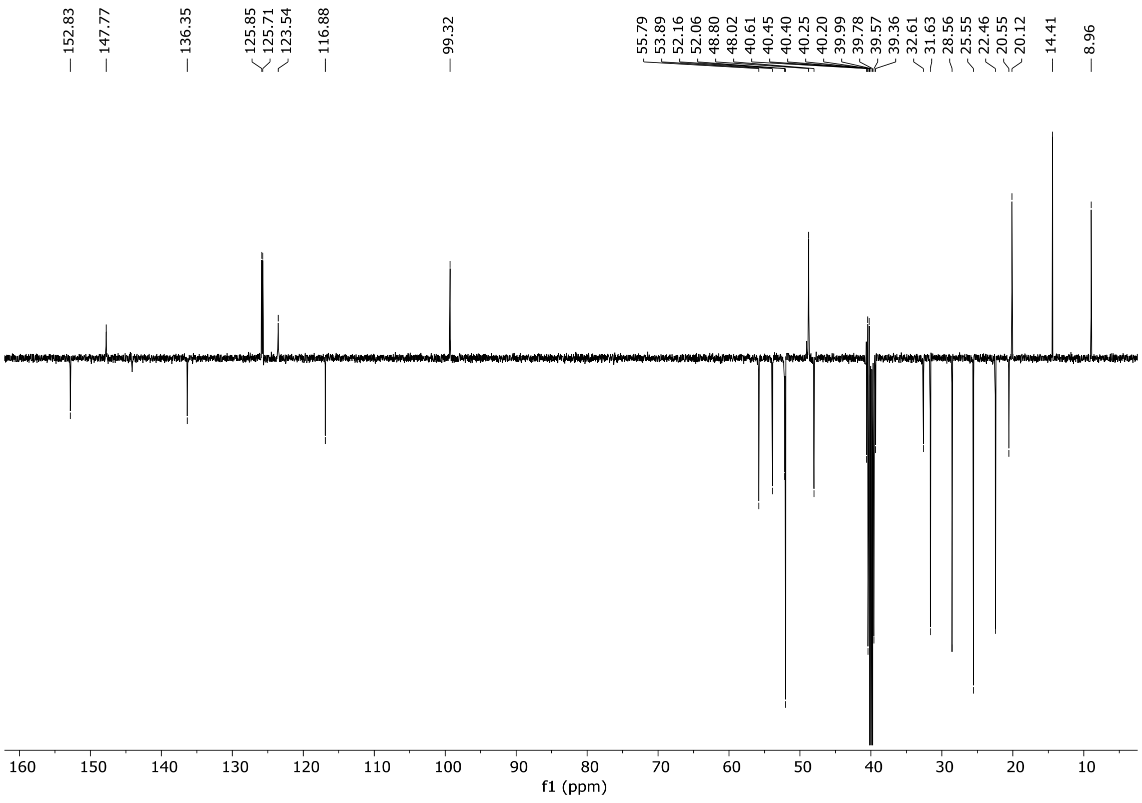

Figure S11. ^{13}C APT NMR spectrum of $[HCQH_2][C_6SO_3]_2$ in DMSO-d_6.

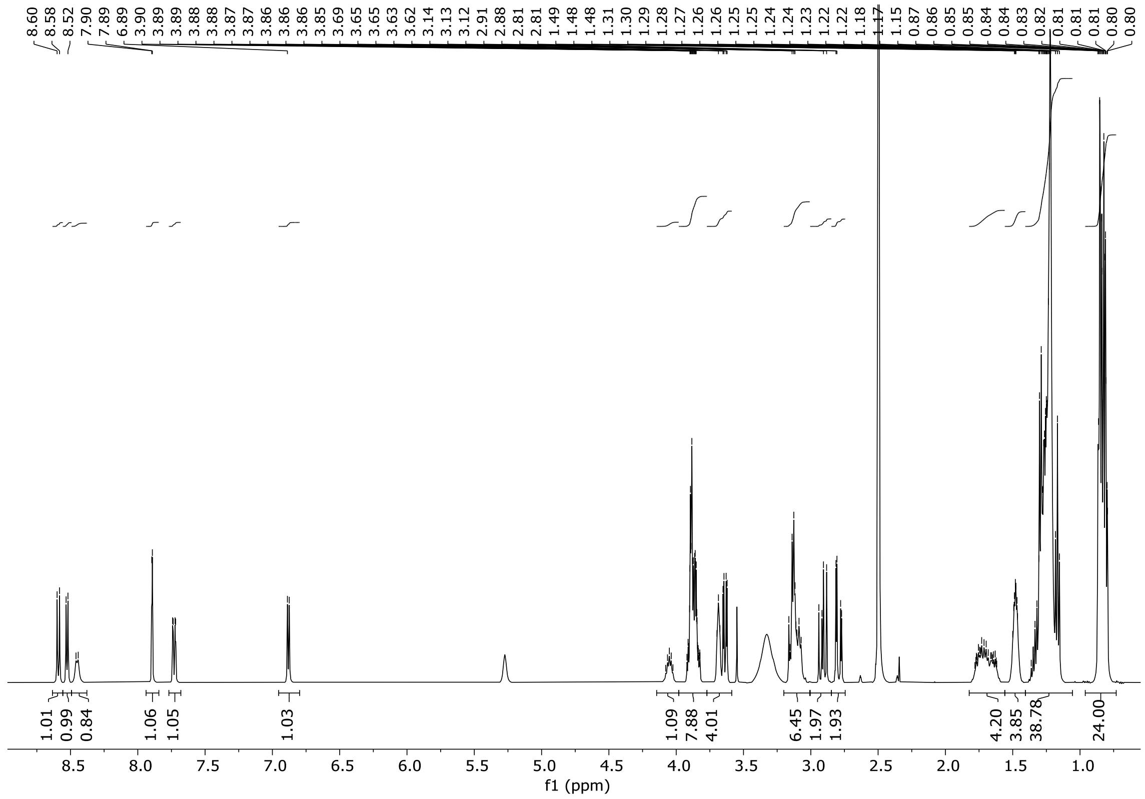

Figure S12. ^{1}H NMR spectrum of $[HCQH_2][DocSO_3]_2$ in DMSO-d_6.

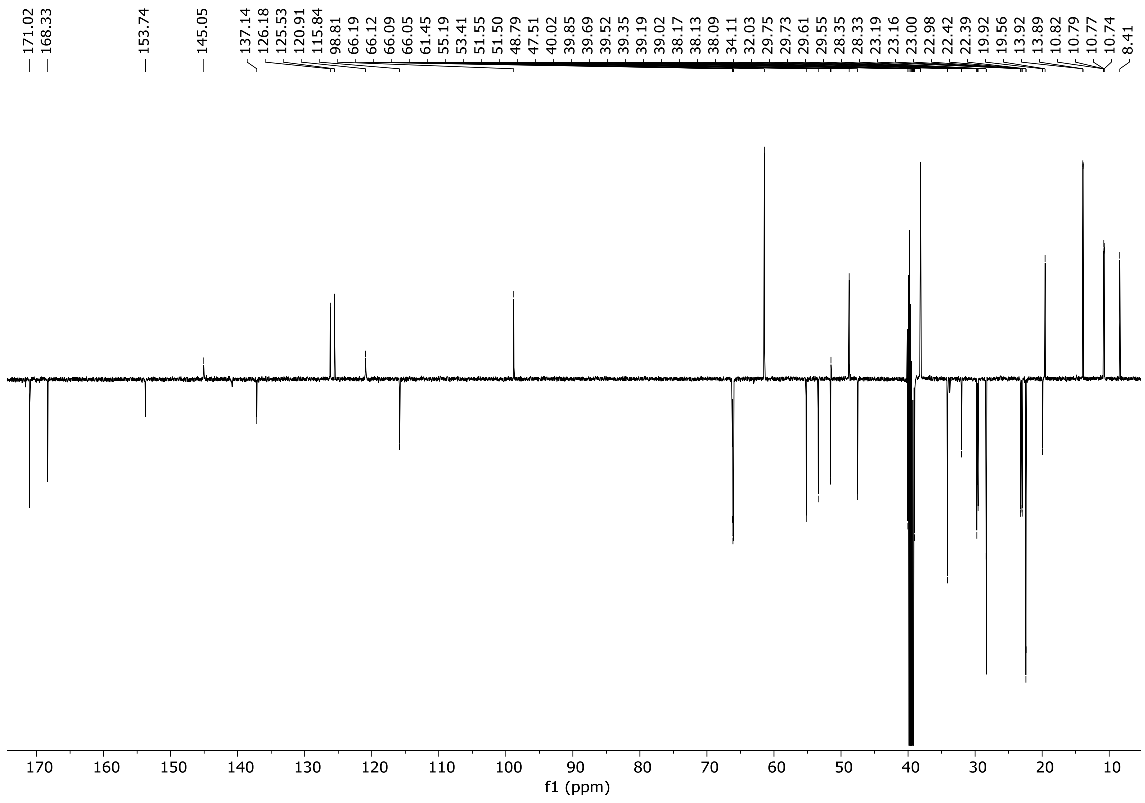

Figure S13. ^{13}C APT NMR spectrum of $[HCQH_2][DocSO_3]_2$ in DMSO-d_6.

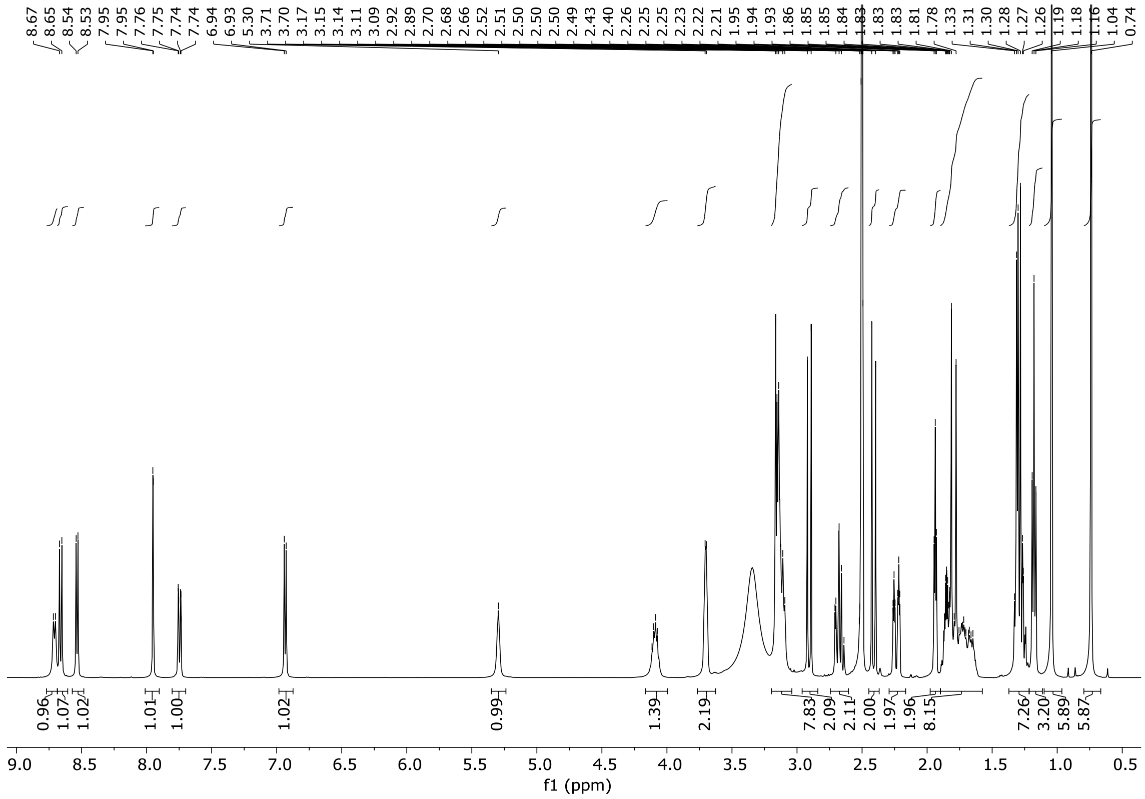

Figure S14. ^1H NMR spectrum of $[HCQH_2][CampSO_3]_2$ in DMSO-d_6.

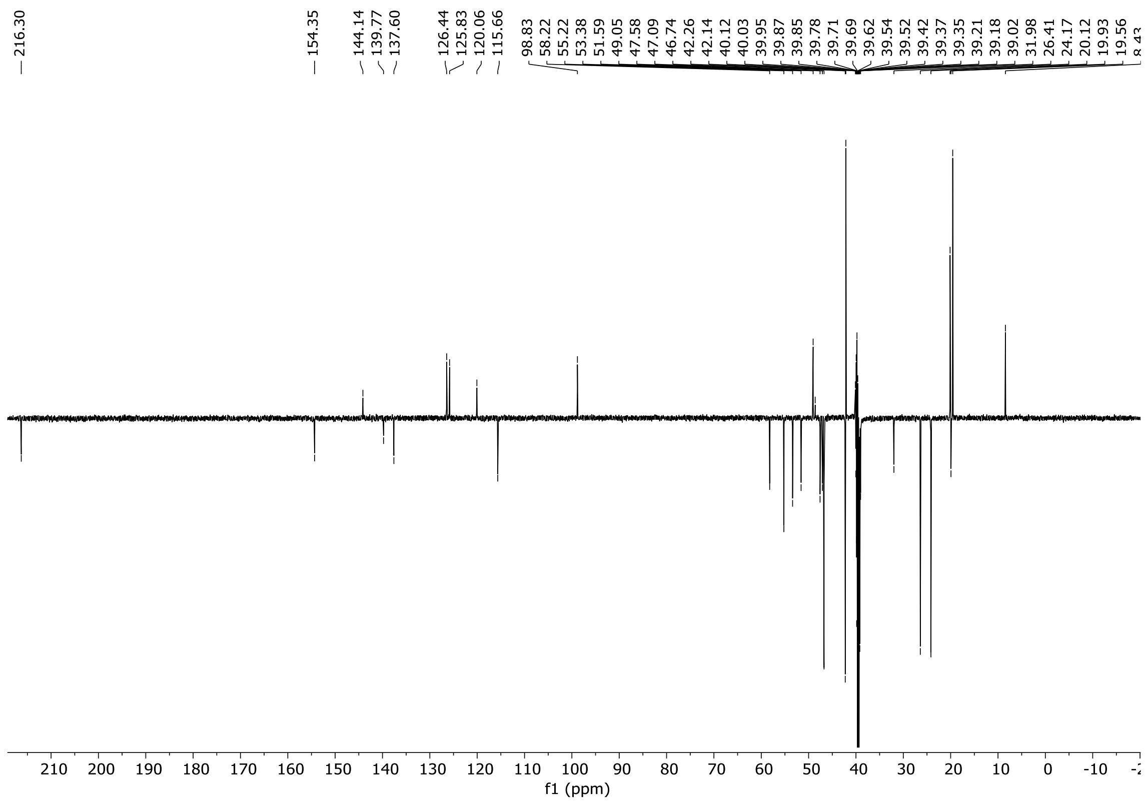

Figure S15. ^{13}C APT NMR spectrum of $[HCQH_2][CampSO_3]_2$ in DMSO-d_6.

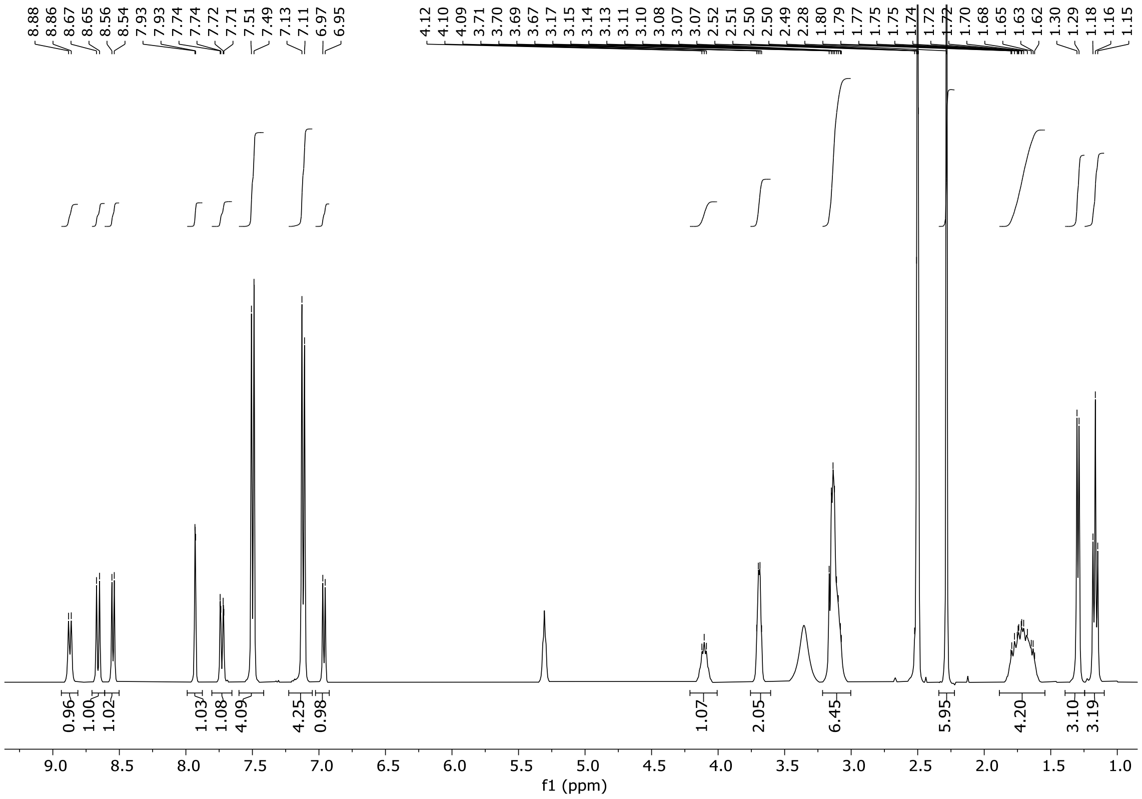

Figure S16. ^{1}H NMR spectrum of $[HCQH_2][p\text{-}TolSO_3]_2$ in DMSO-d_6.

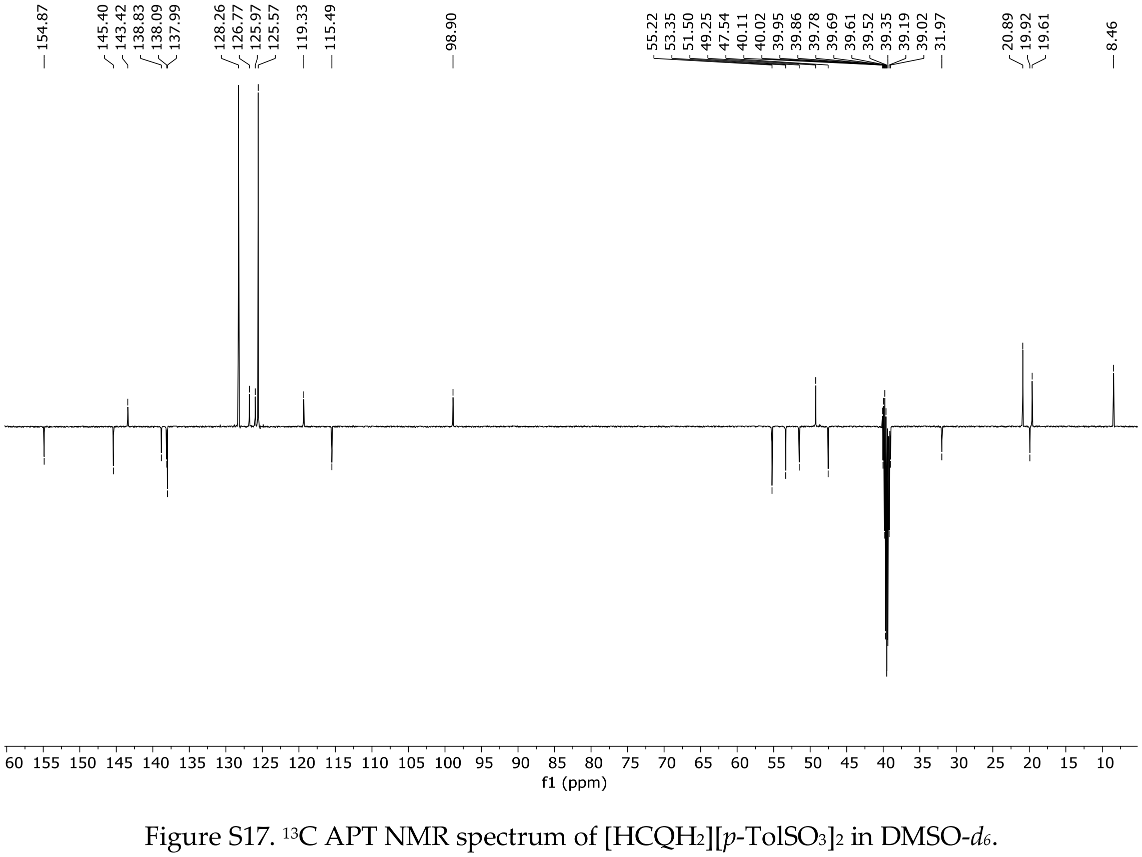

Figure S17. ^{13}C APT NMR spectrum of $[HCQH_2][p\text{-}TolSO_3]_2$ in DMSO-d_6.

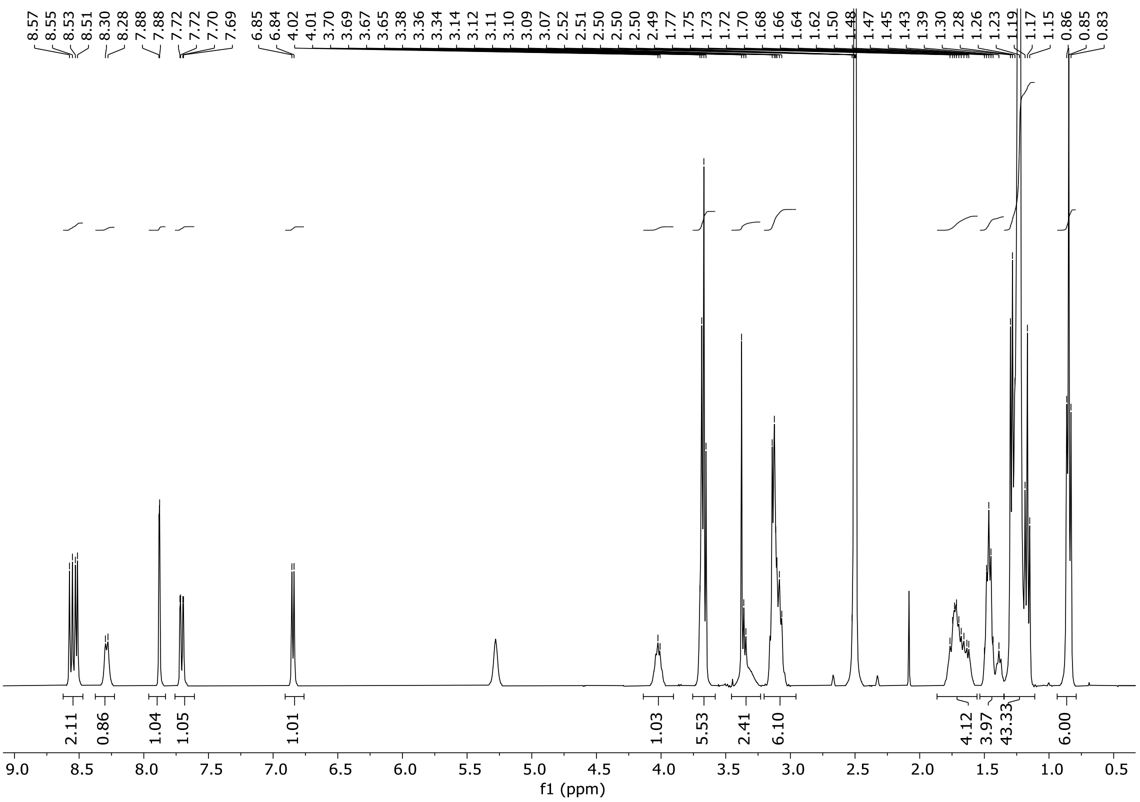

Figure S18. ^{1}H NMR spectrum of $[HCQH_2][C_{12}SO_4]_2$ in DMSO-d_6.

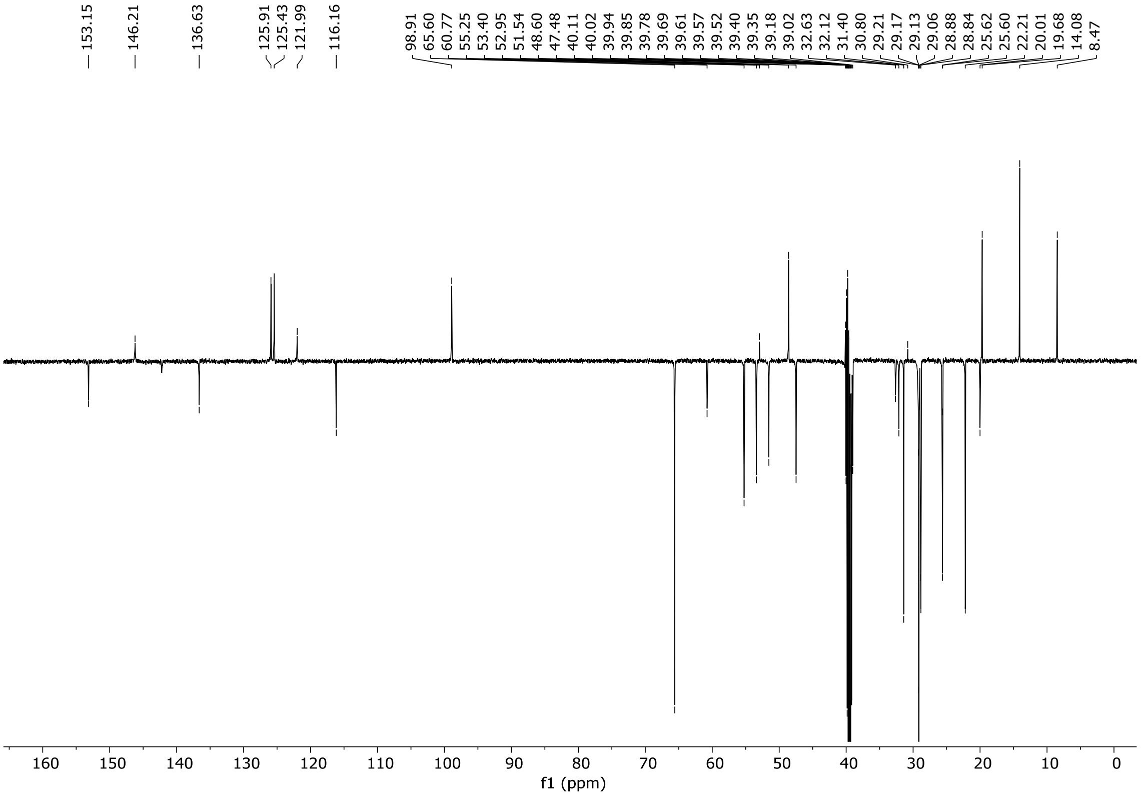

Figure S19. ^{13}C APT NMR spectrum of $[HCQH_2][C_{12}SO_4]_2$ in DMSO-d_6.

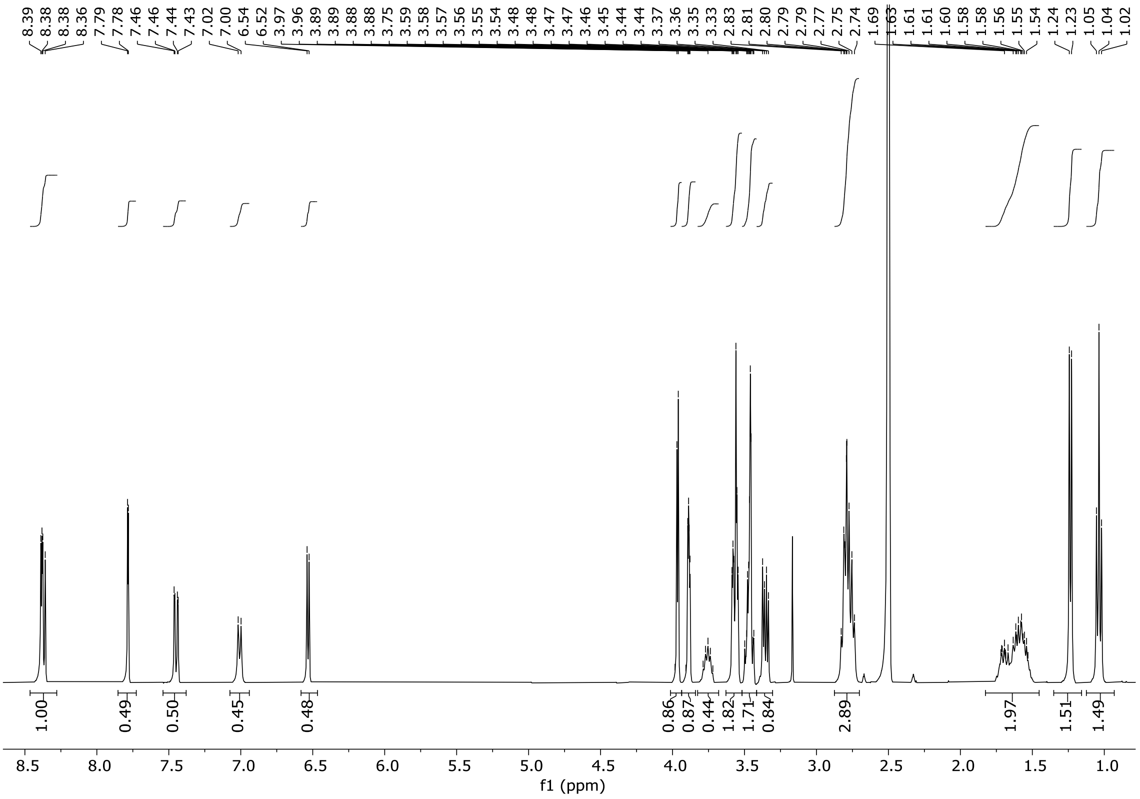

Figure S20. ^{1}H NMR spectrum of $[HCQH_2][GlcCOO]_2$ in DMSO-d_6.

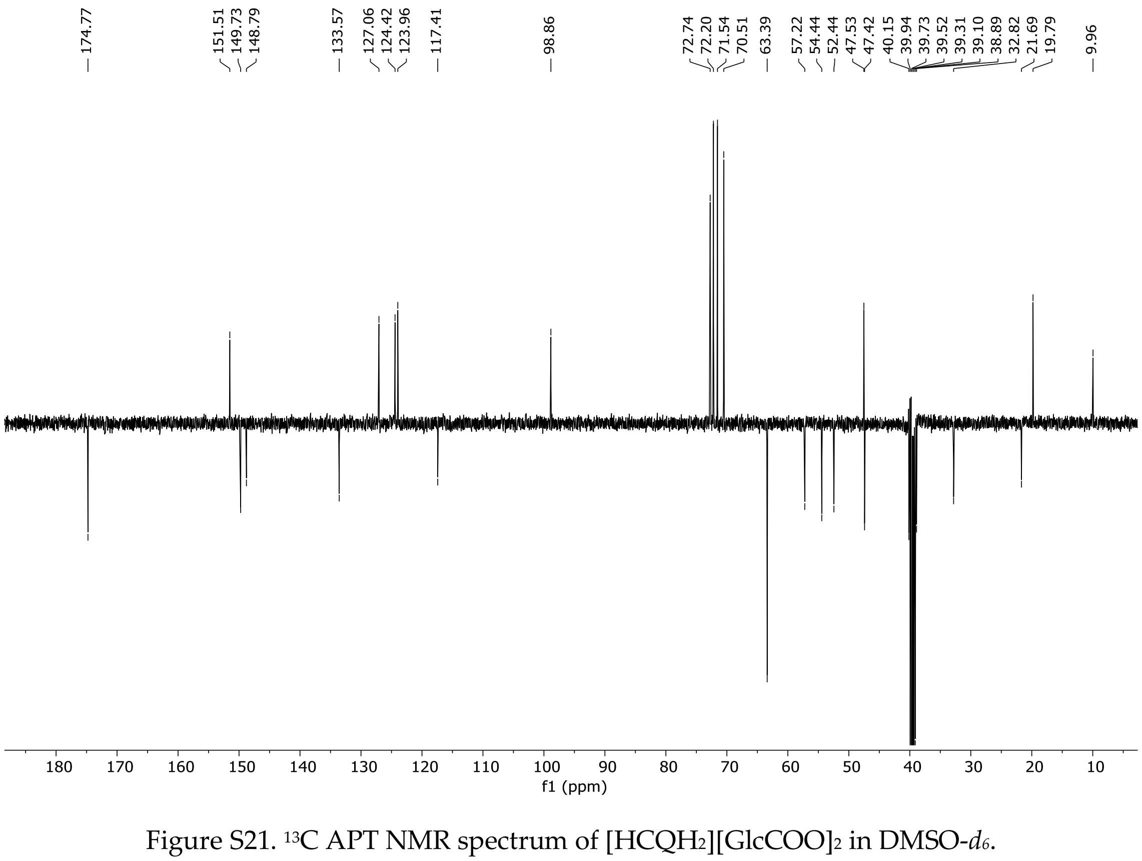

Figure S21. ^{13}C APT NMR spectrum of $[HCQH_2][GlcCOO]_2$ in DMSO-d_6.

HRMS spectra

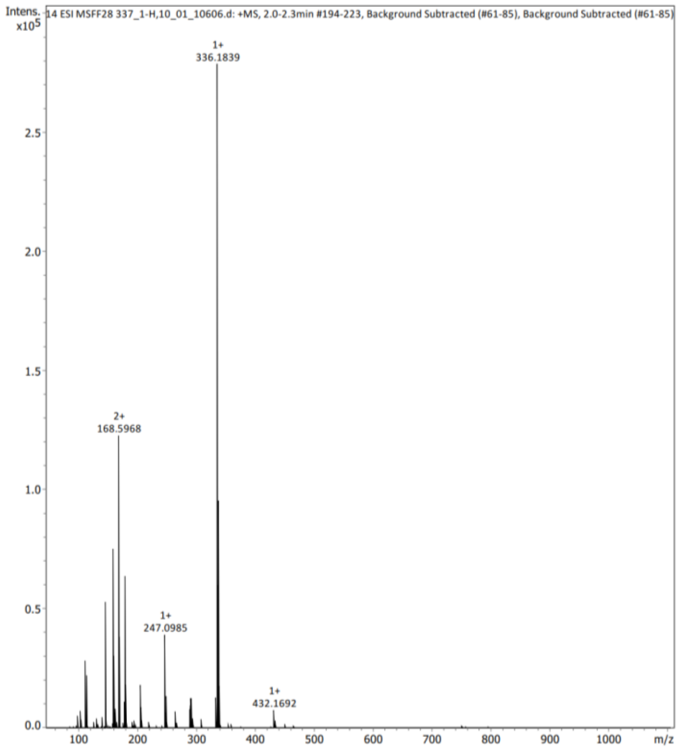

Figure S22. ESI-TOF mass spectra in positive mode of $[HCQH_2][C_1SO_3]_2$.

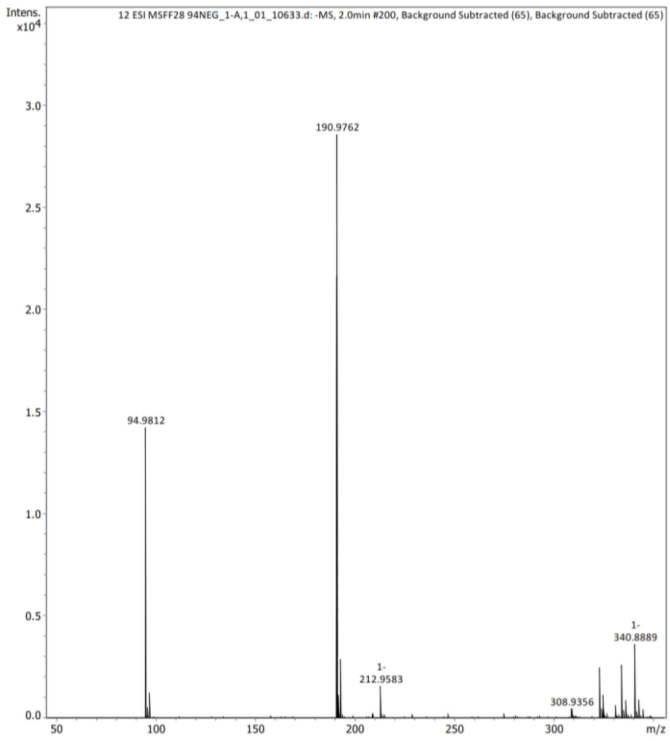

Figure S23. ESI-TOF mass spectra in negative mode of $[HCQH_2][C_1SO_3]_2$.

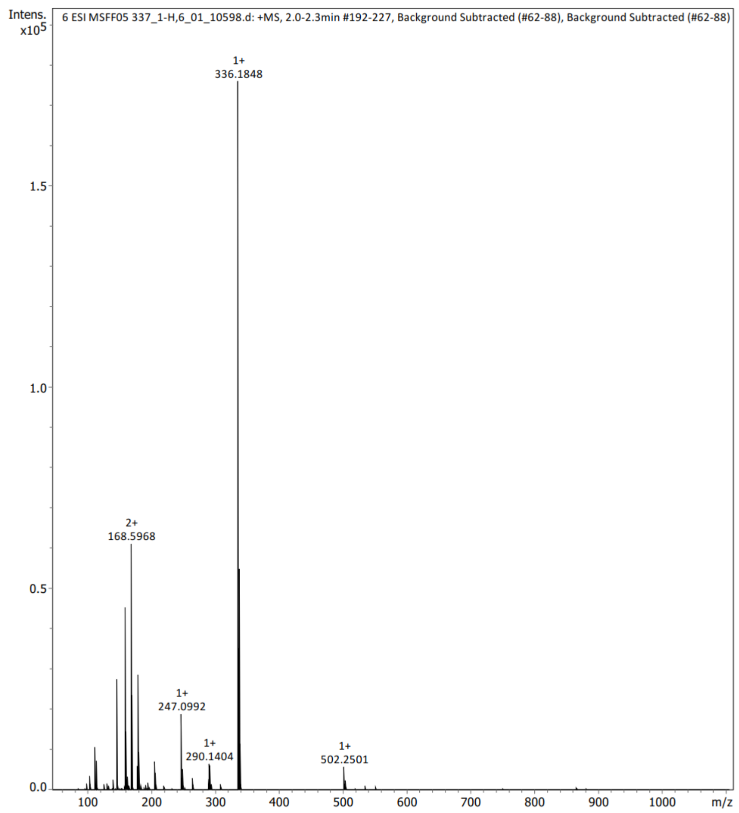

Figure S24. ESI-TOF mass spectra in positive mode of $[HCQH_2][C_6SO_3]_2$.

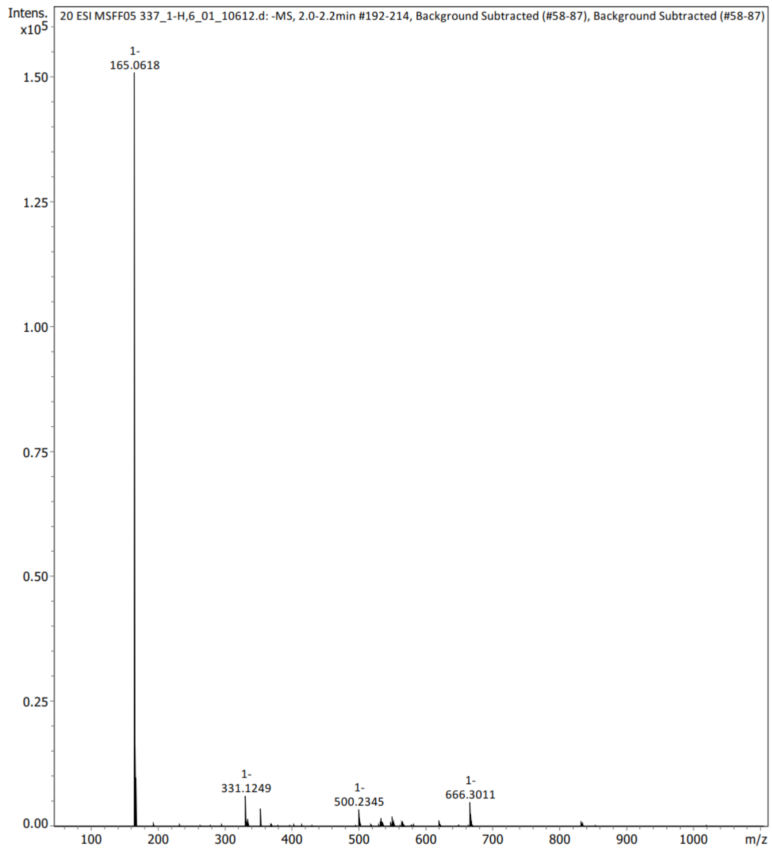

Figure S25. ESI-TOF mass spectra in negative mode of $[HCQH_2][C_6SO_3]_2$.

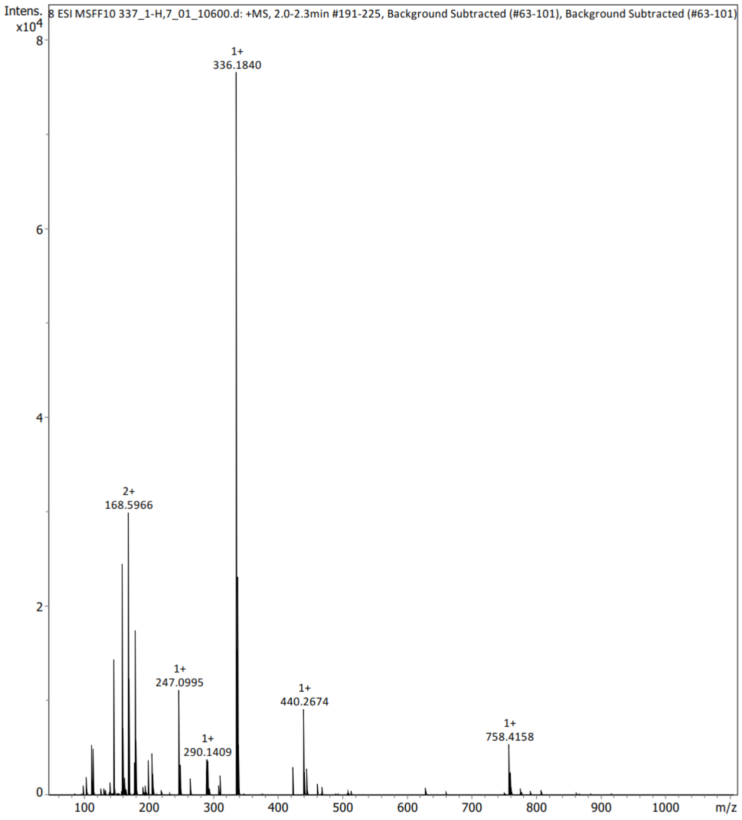

Figure S26. ESI-TOF mass spectra in positive mode of $[HCQH_2][DocSO_3]_2$.

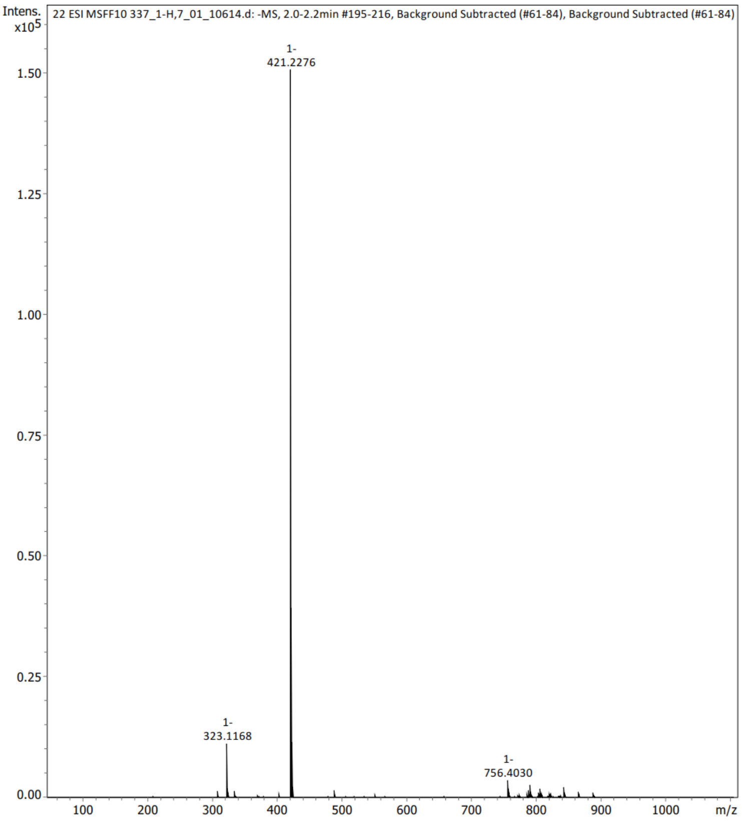

Figure S27. ESI-TOF mass spectra in negative mode of $[HCQH_2][DocSO_3]_2$.

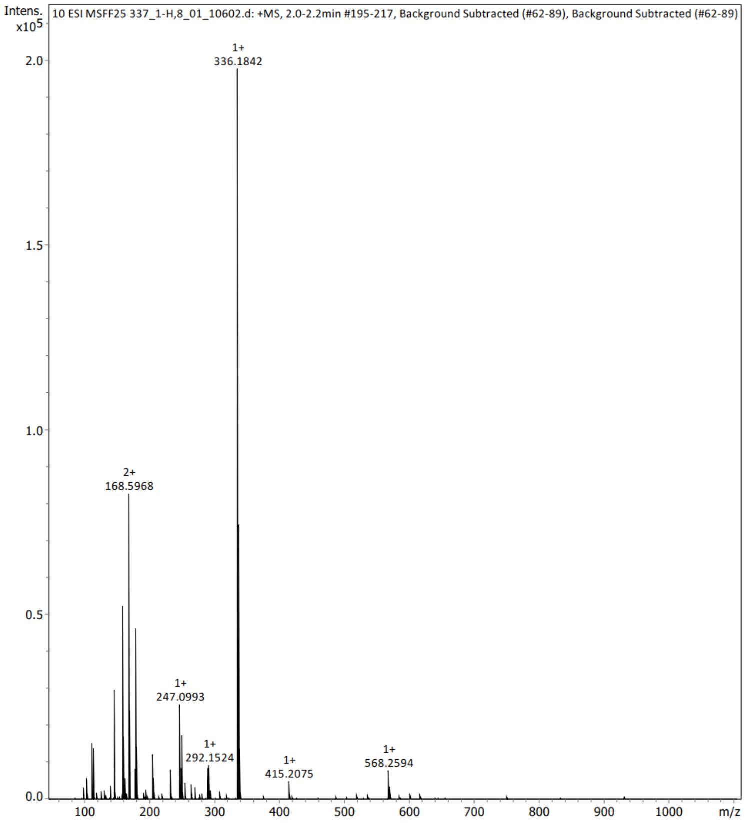

Figure S28. ESI-TOF mass spectra in positive mode of $[HCQH_2][CampSO_3]_2$.

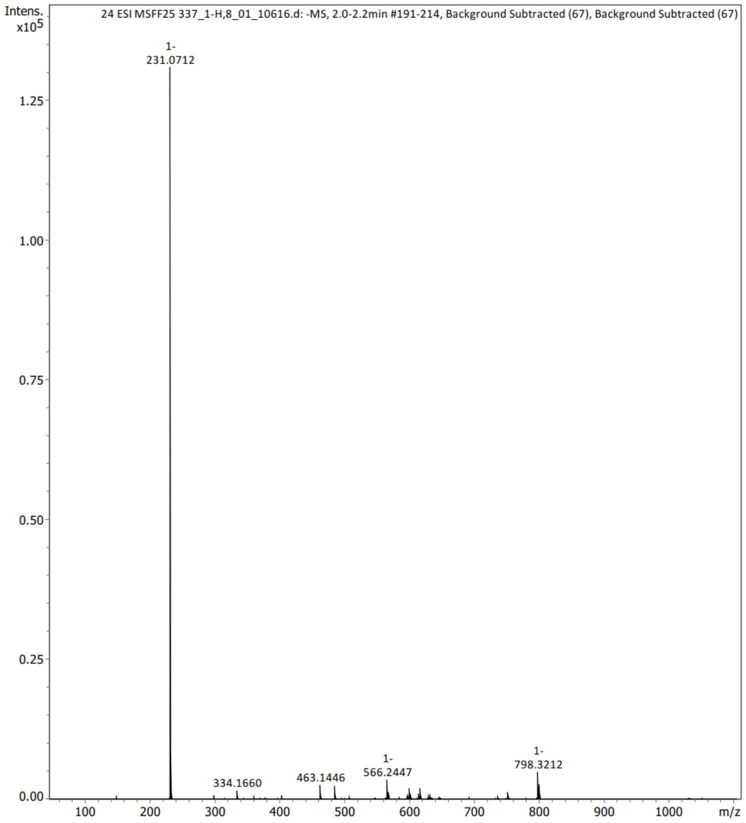

Figure S29. ESI-TOF mass spectra in negative mode of $[HCQH_2][CampSO_3]_2$.

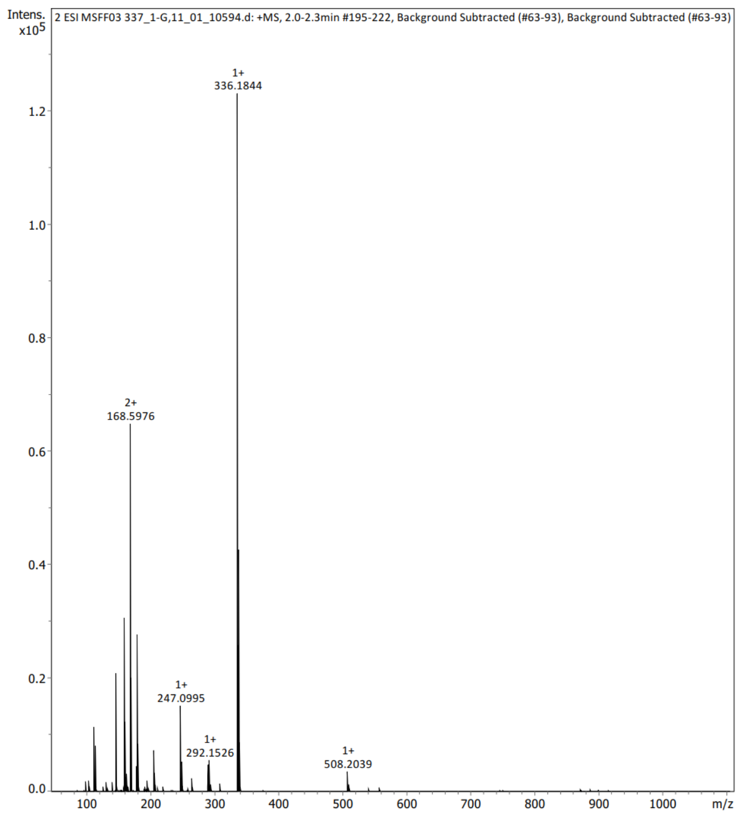

Figure S30. ESI-TOF mass spectra in positive mode of $[HCQH_2][p\text{-}TolSO_3]_2$.

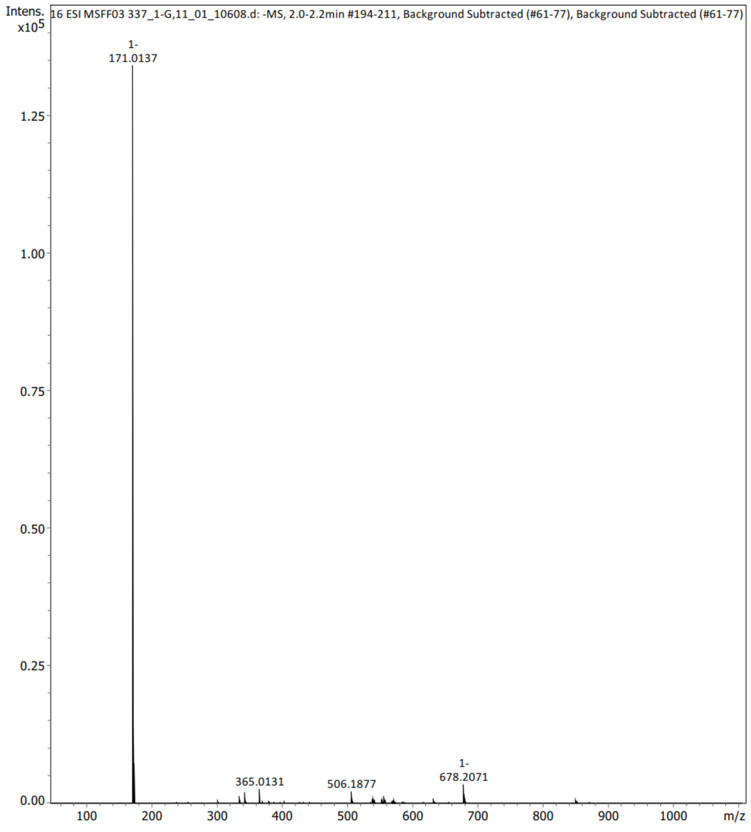

Figure S31. ESI-TOF mass spectra in negative mode of $[HCQH_2][p\text{-}TolSO_3]_2$.

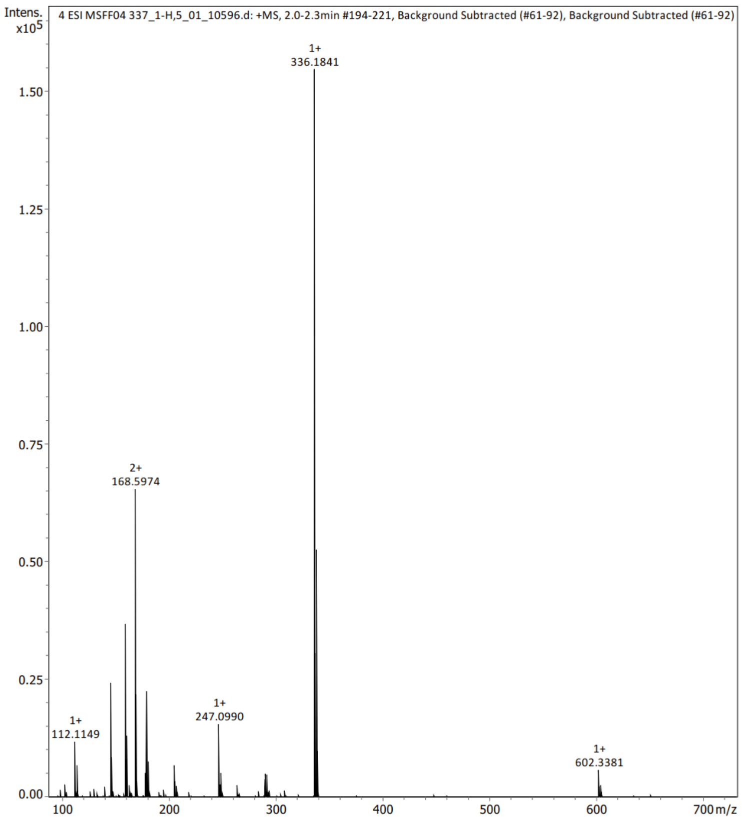

Figure S32. ESI-TOF mass spectra in positive mode of $[HCQH_2][C_{12}SO_4]_2$.

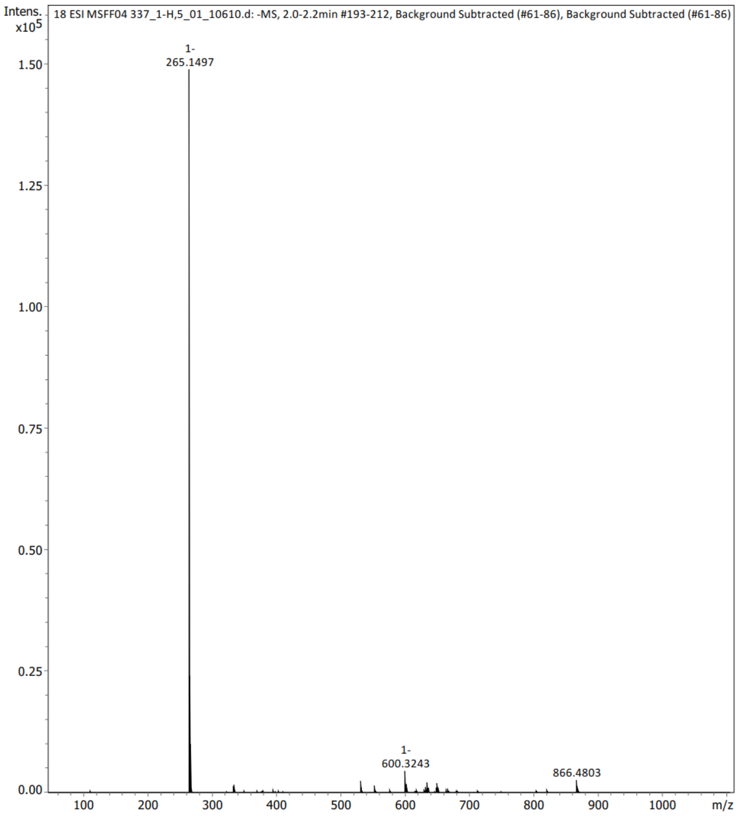

Figure S33. ESI-TOF mass spectra in negative mode of $[HCQH_2][C_{12}SO_4]_2$.

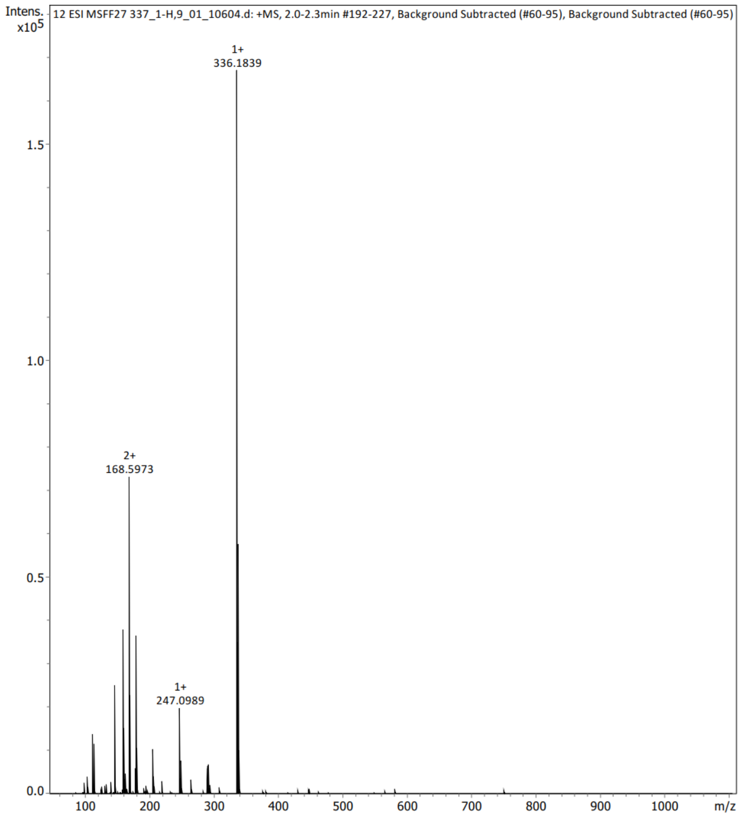

Figure S34. ESI-TOF mass spectra in positive mode of $[HCQH_2][GlcCOO]_2$.

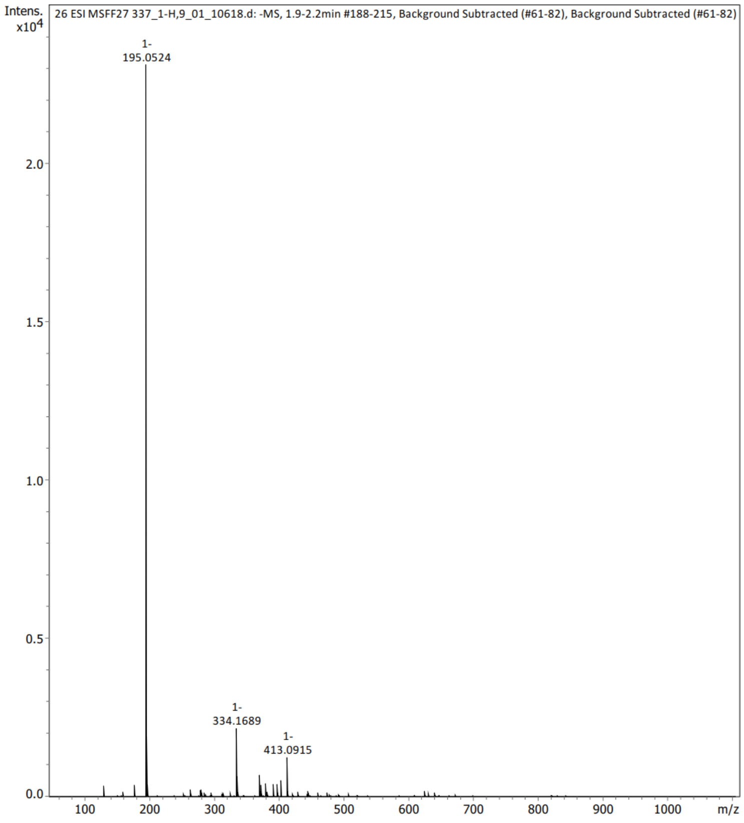

Figure S35. ESI-TOF mass spectra in negative mode of $[HCQH_2][GlcCOO]_2$.

Table S1. Experimental and calculated *m/z* values and corresponding errors (ppm) for the $[M+1]^+$ and $[M+2]^{2+}$ ions in positive mode of ESI-TOF for each HCQ-IL.

	**Exp. *m/z* $[M+1]^+$**	**Calc. *m/z* $[M+1]^+$**	**error /ppm**	**Exp. *m/z* $[M+2]^{2+}$**	**Calc. *m/z* $[M+2]^{2+}$**	**error /ppm**
$[HCQH_2][C_1SO_3]_2$	336.1839	336.1837	-0.59	168.5968	168.5955	-7.71
$[HCQH_2][C_6SO_3]_2$	336.1848	336.1837	-3.27	168.5968	168.5955	-7.71
$[HCQH_2][DocSO_3]_2$	336.1840	336.1837	-0.89	168.5966	168.5955	-6.52
$[HCQH_2][CampSO_3]_2$	336.1842	336.1837	-1.49	168.5968	168.5955	-7.71
$[HCQH_2][p\text{-}TolSO_3]_2$	336.1844	336.1837	-2.08	168.5976	168.5955	-12.46
$[HCQH_2][C_{12}SO_4]_2$	336.1841	336.1837	-1.19	168.5974	168.5955	-11.27
$[HCQH_2][GlcCOO]_2$	336.1839	336.1837	-0.59	168.5973	168.5955	-10.68

Table S2. Experimental and calculated *m/z* values and corresponding errors (ppm) for the $[M-1]^-$ ion in negative mode of ESI-TOF for each HCQ-IL.

	**Exp. *m/z* $[M-1]^-$**	**Calc. *m/z* $[M-1]^-$**	**error /ppm**
$[HCQH_2][C_1SO_3]_2$	94.9808	94.9812	4.21
$[HCQH_2][C_6SO_3]_2$	165.0618	165.0590	-16.96
$[HCQH_2][DocSO_3]_2$	421.2276	421.2265	-2.61
$[HCQH_2][CampSO_3]_2$	231.0696	231.0712	6.92
$[HCQH_2][p\text{-}TolSO_3]_2$	171.0137	171.0121	-9.36
$[HCQH_2][C_{12}SO_4]_2$	265.1497	265.1479	-6.79
$[HCQH_2][GlcCOO]_2$	195.0510	195.0524	7.18

DSC thermograms

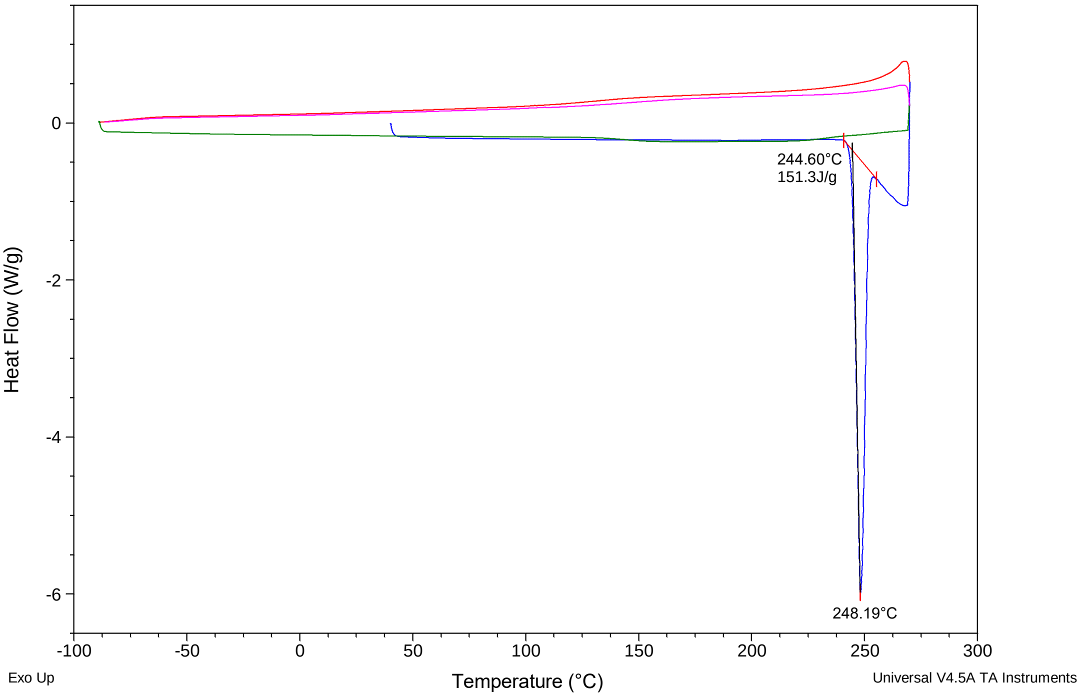

Figure S36. DSC thermogram of $[HCQH_2][SO_4]$.

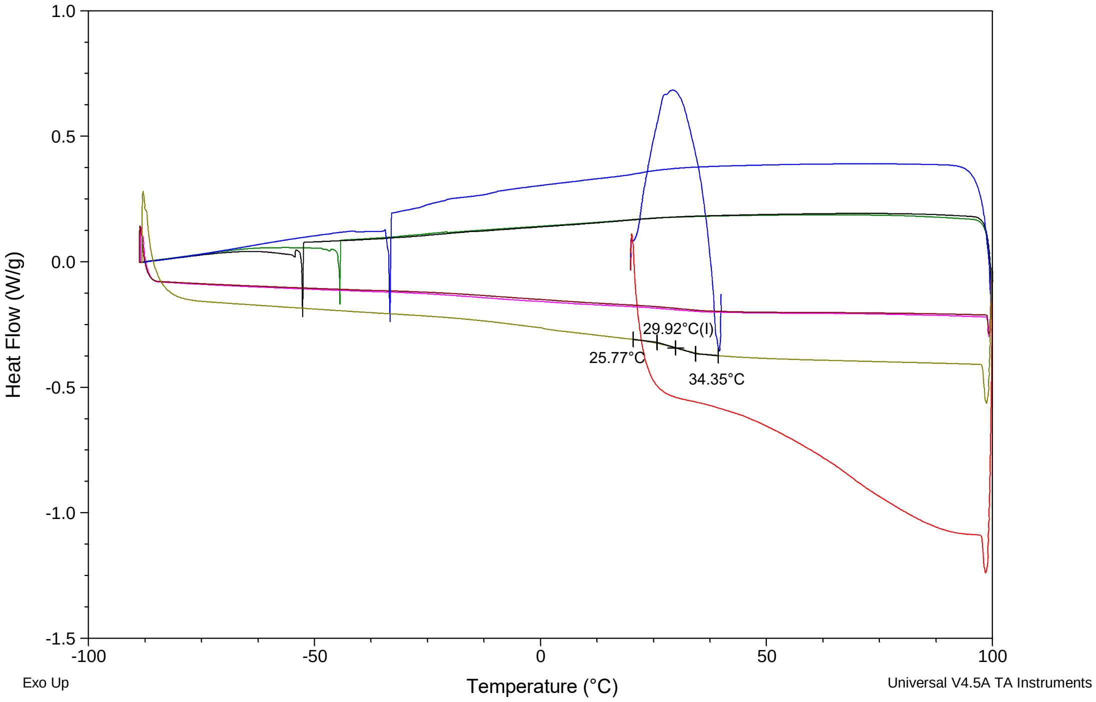

Figure S37. DSC thermogram of $[HCQH_2][C_1SO_3]_2$.

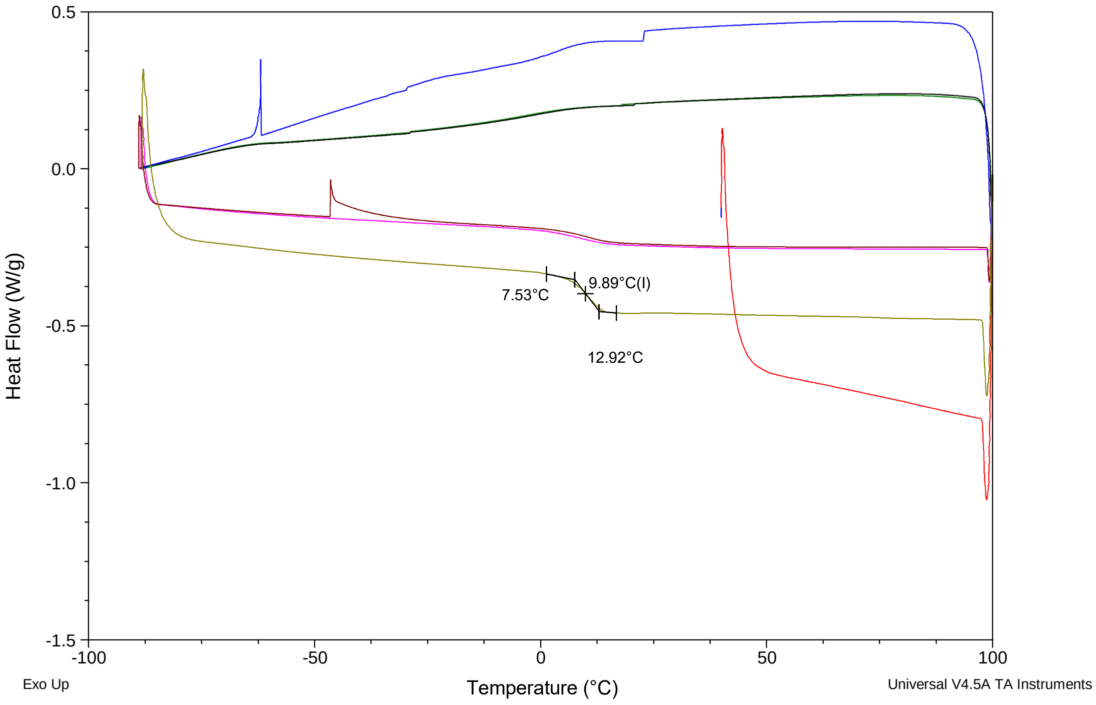

Figure S38. DSC thermogram of [HCQH2][C6SO3]2.

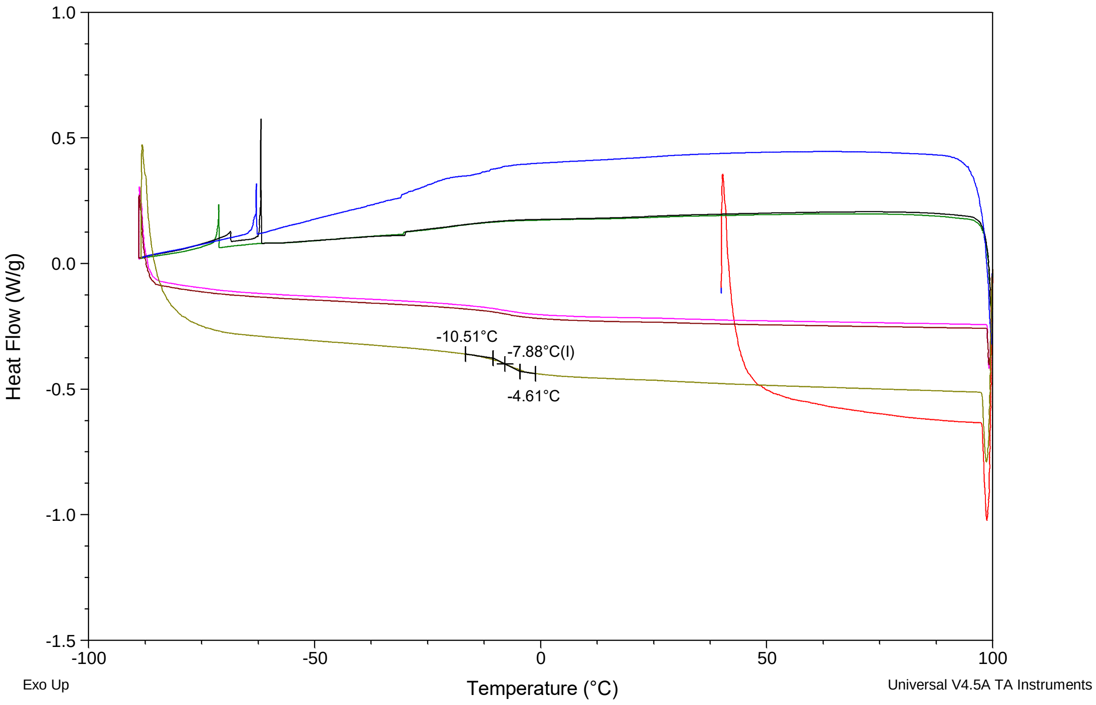

Figure S39. DSC thermogram of [HCQH2][DocSO3]2.

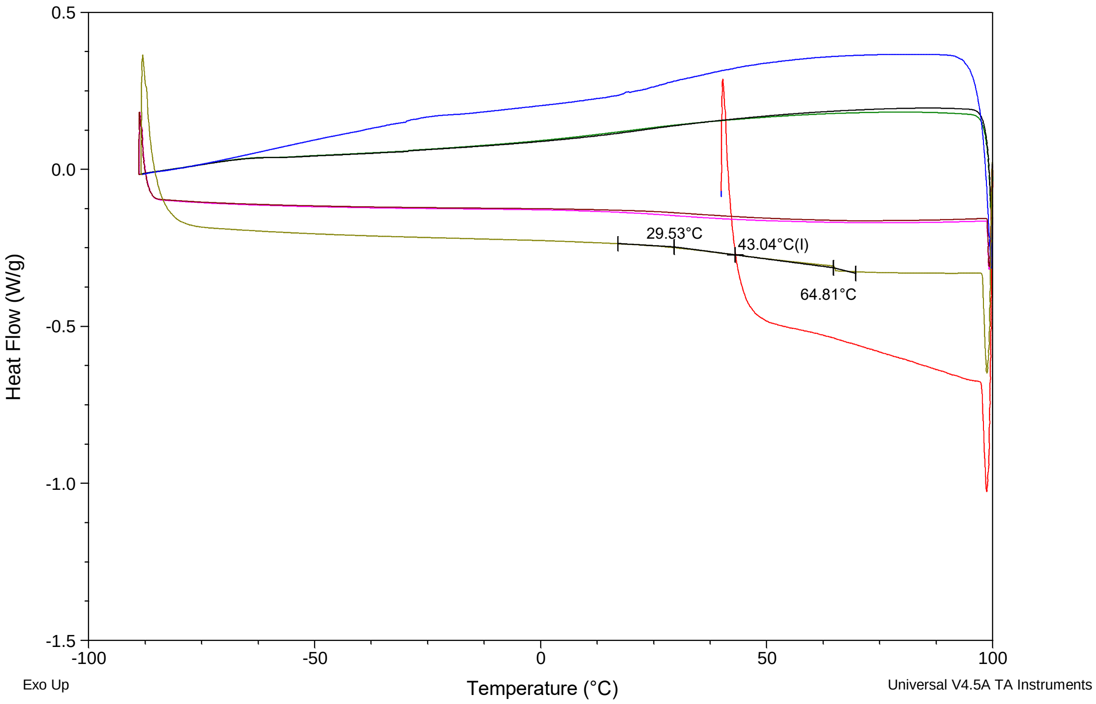

Figure S40. DSC thermogram of [HCQH2][CampSO3]2.

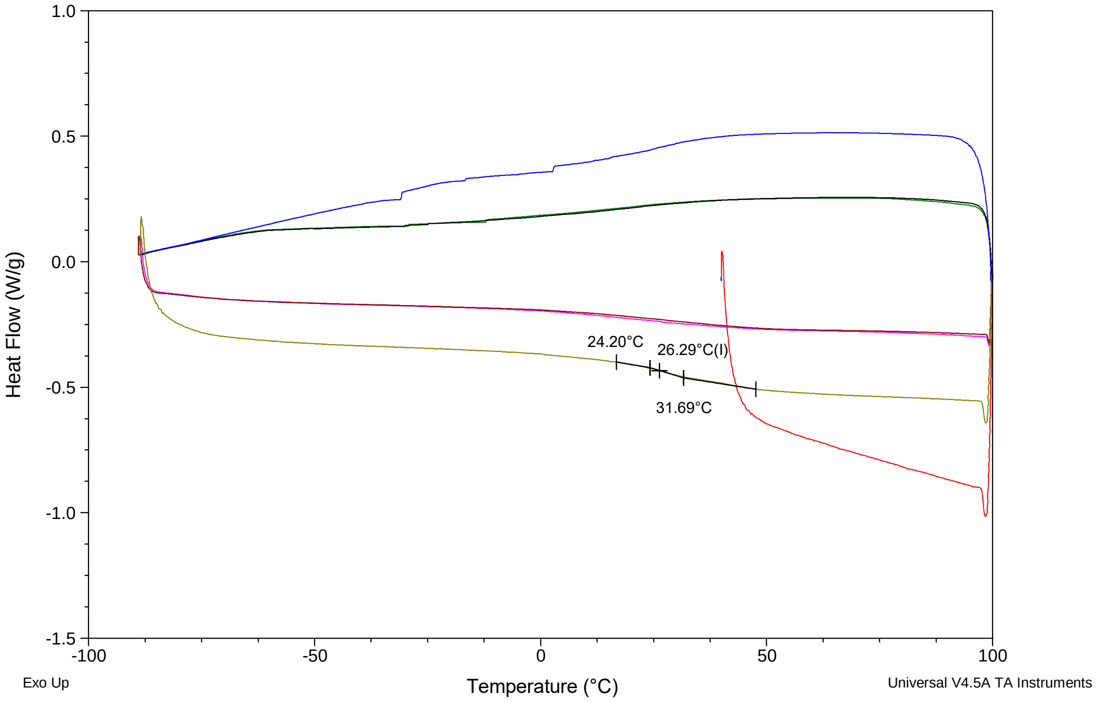

Figure S41. DSC thermogram of [HCQH2][*p*-TolSO3]2.

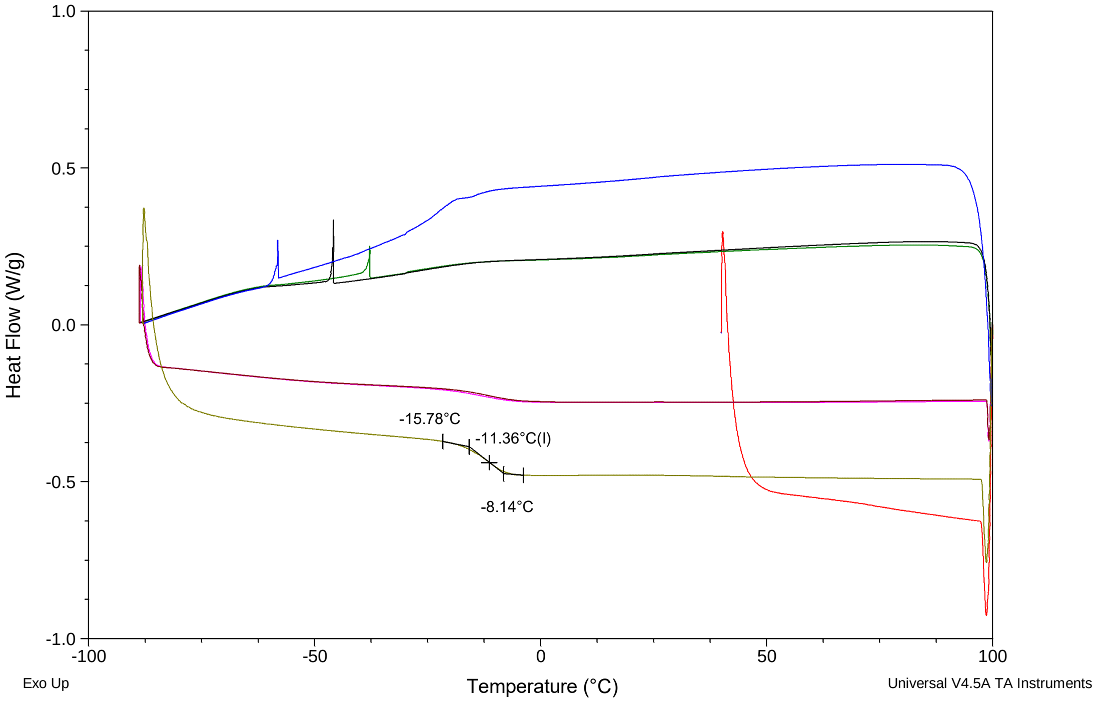

Figure S42. DSC thermogram of [HCQH2][C12SO4]2.

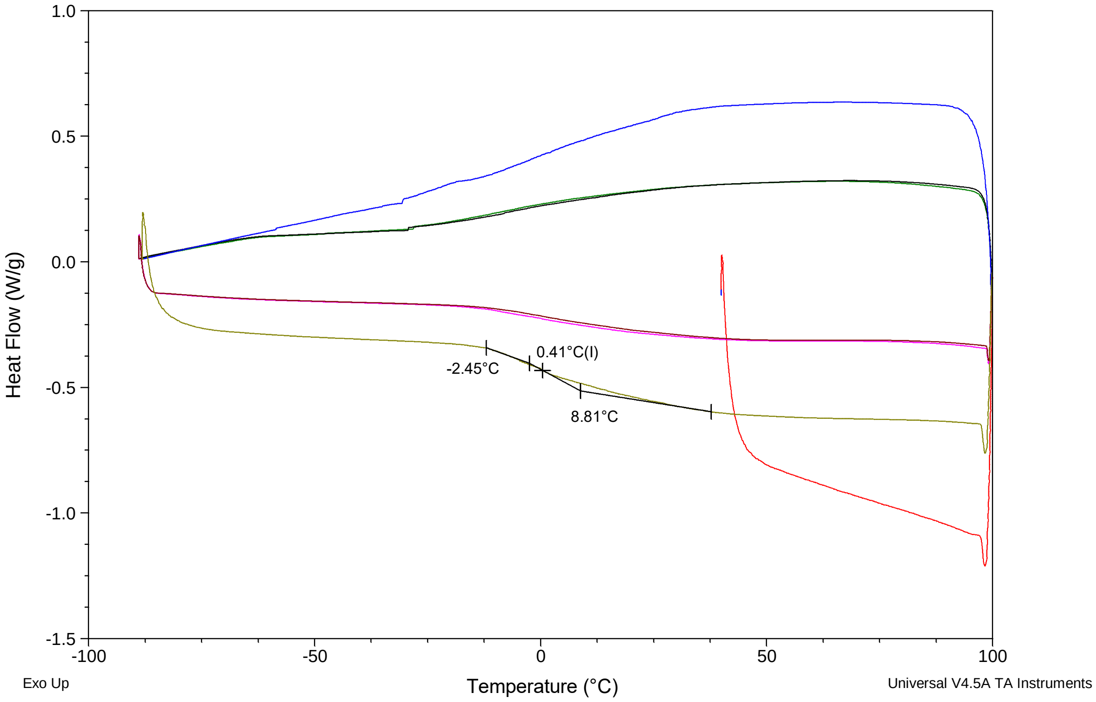

Figure S43. DSC thermogram of [HCQH2][GlcCOO]2.

Critical micelle concentrations

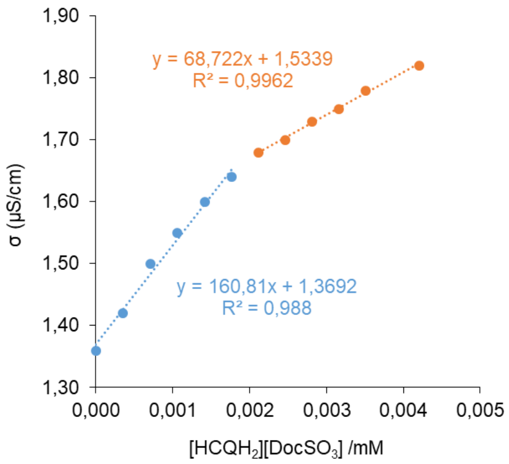

Figure S44. Plot of conductivity *vs* concentration of $[HCQH_2][DocSO_3]_2$ (**5**).

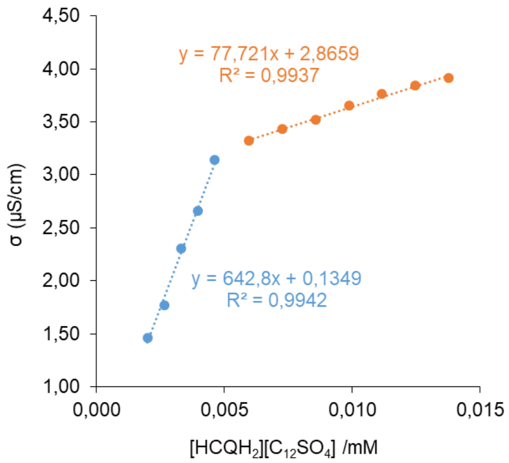

Figure S45. Plot of conductivity *vs* concentration of $[HCQH_2][C_{12}SO_4]_2$ (**8**).
